# Host-Microbe Interactions in the Chemosynthetic *Riftia pachyptila* Symbiosis

**DOI:** 10.1128/mBio.02243-19

**Published:** 2019-12-17

**Authors:** Tjorven Hinzke, Manuel Kleiner, Corinna Breusing, Horst Felbeck, Robert Häsler, Stefan M. Sievert, Rabea Schlüter, Philip Rosenstiel, Thorsten B. H. Reusch, Thomas Schweder, Stephanie Markert

**Affiliations:** a Institute of Marine Biotechnology e.V., Greifswald, Germany; b Institute of Pharmacy, Department of Pharmaceutical Biotechnology, University of Greifswald, Greifswald, Germany; c Energy Bioengineering Group, University of Calgary, Calgary, Canada; d Department of Plant & Microbial Biology, North Carolina State University, Raleigh, North Carolina, USA; e Monterey Bay Aquarium Research Institute, Moss Landing, California, USA; f Scripps Institution of Oceanography, University of California San Diego, San Diego, California, USA; g Institute of Clinical Molecular Biology (IKMB), Kiel University, Kiel, Germany; h Biology Department, Woods Hole Oceanographic Institution, Woods Hole, Massachusetts, USA; i Imaging Center of the Department of Biology, University of Greifswald, Greifswald, Germany; j Marine Evolutionary Ecology, GEOMAR Helmholtz Centre for Ocean Research Kiel, Kiel, Germany; Northeastern University; University of Hawaii at Manoa

**Keywords:** host-microbe interactions, symbiosis, holobiont, chemosynthesis, hydrothermal vents, metaproteomics

## Abstract

The deep-sea tubeworm Riftia pachyptila lacks a digestive system but completely relies on bacterial endosymbionts for nutrition. Although the symbiont has been studied in detail on the molecular level, such analyses were unavailable for the animal host, because sequence information was lacking. To identify host-symbiont interaction mechanisms, we therefore sequenced the *Riftia* transcriptome, which served as a basis for comparative metaproteomic analyses of symbiont-containing versus symbiont-free tissues, both under energy-rich and energy-limited conditions. Our results suggest that metabolic interactions include nutrient allocation from symbiont to host by symbiont digestion and substrate transfer to the symbiont by abundant host proteins. We furthermore propose that *Riftia* maintains its symbiont by protecting the bacteria from oxidative damage while also exerting symbiont population control. Eukaryote-like symbiont proteins might facilitate intracellular symbiont persistence. Energy limitation apparently leads to reduced symbiont biomass and increased symbiont digestion. Our study provides unprecedented insights into host-microbe interactions that shape this highly efficient symbiosis.

## Abstract (precis)

All animals are associated with microorganisms; hence, host-microbe interactions are of fundamental importance for life on earth. However, we know little about the molecular basis of these interactions. Therefore, we studied the deep-sea *Riftia pachyptila* symbiosis, a model association in which the tubeworm host is associated with only one phylotype of endosymbiotic bacteria and completely depends on this sulfur-oxidizing symbiont for nutrition. Using a metaproteomics approach, we identified both metabolic interaction processes, such as substrate transfer between the two partners, and interactions that serve to maintain the symbiotic balance, e.g., host efforts to control the symbiont population or symbiont strategies to modulate these host efforts. We suggest that these interactions are essential principles of mutualistic animal-microbe associations.

## INTRODUCTION

All animals are associated with microorganisms (1–3), and consequently, mutualistic bacterium-animal symbioses play critical roles in the physiology, ecology, and evolution of animals, thereby shaping life on our planet. Many of these mutualistic symbioses are based on nutritional benefits for both partners. Symbionts supply their host with nutrients otherwise lacking in the host's diet, while the host in turn provides the symbionts with metabolites, shelter, and optimal growth conditions (4). To establish and stably maintain their alliance, the partners have to interact on the molecular level. The host's immune system needs to control the symbiont population without erasing it altogether (5), for example, by restricting the symbionts to certain organs and/or by downregulating its own immune response (reviewed in reference 6). Symbionts, on the other hand, often employ strategies resembling those of pathogens to colonize and persist in their host. For example, similar protein secretion systems are used by both symbionts and pathogens for interactions with the host (4, 7–9).

In many animals, host-microbe interactions are difficult to assess due to the high number of microbes potentially involved and the presence of long- and short-term associations, which are hard to distinguish (9). Therefore, low-complexity models are important to identify and characterize interaction mechanisms (10). Symbioses of marine invertebrates and their chemoautotrophic symbionts have emerged as suitable study systems. In these symbioses, animal hosts such as gutless annelids and bivalves are often tightly associated with one or a few symbiont types, which enable the eukaryotes to prevail in otherwise hostile environments (11). One of the most conspicuous representatives of these associations, and the first animal in which chemoautotrophic symbionts were discovered, is the giant tube worm *Riftia pachyptila* (short *Riftia*), which thrives around deep-sea hydrothermal vents of the East Pacific (12, 13). The host’s absolute dependency on its symbiont makes *Riftia* an ideal system to study beneficial host-microbe interactions in a mutualistic symbiosis.

The worm completely lacks a digestive system but instead receives all necessary nutrients from its chemosynthetic endosymbiont (12–15). The host in turn provides the endosymbiont with all necessary inorganic compounds for chemosynthesis (16). This association is remarkably productive: *Riftia* grows extraordinarily fast (>85-cm increase in tube length per year [17]) and reaches body lengths of up to 1.5 m (18).

The uncultured gammaproteobacterial *Riftia* symbiont, a single 16S rRNA phylotype tentatively named “*Candidatus* Endoriftia persephone” (19–21), densely populates bacteriocytes in the host trophosome, a specialized organ that fills most of the worm’s body cavity (14). The bacteria oxidize inorganic reduced compounds, such as hydrogen sulfide, to generate energy for carbon fixation (13, 22–26). The symbiont can store elemental sulfur, an intermediate of sulfide oxidation, in sulfur globules (27). Trophosome tissue containing large amounts of stored sulfur has a light yellowish color. During sulfide limitation, i.e., when energy availability is restricted due to low environmental sulfide concentrations, stored sulfur is consumed and the trophosome appears much darker (27–29). Thus, the energetic status of the symbiosis can be directly inferred from the color of the trophosome.

*Riftia* has been extensively studied, especially with respect to its anatomy, biochemistry, symbiont transmission, and substrate transfer between host, symbionts, and the environment (for examples, see references 24 and 29–32; see references 16 and 33 for reviews). The symbiont’s metabolism has been studied in detail as well (16), in particular by means of metagenomics and metaproteomics (19, 25, 34, 35). However, little is known about interactions between the two symbiotic partners and, particularly, about the proteins directly involved in these processes.

Our study aimed to illuminate the underlying mechanisms of host-symbiont interactions on the protein level. For this purpose, we employed a state-of-the-art global metaproteomics approach, which required comprehensive sequence data for both partners. While the genome of the *Riftia* symbiont was sequenced previously (19, 34), until now no such information was available for the host. Therefore, we sequenced the transcriptome of the *Riftia* host *de novo*. This enabled us to build a comprehensive protein database, which we used to compare protein abundance patterns in symbiont-containing and symbiont-free *Riftia* tissues. By comparing sulfur-rich and sulfur-depleted specimens, we furthermore examined the dynamics of host-symbiont interactions under high- and low-energy conditions. Our analysis sheds light on metabolite exchange processes between both partners, on the host’s symbiont maintenance strategies, and on the symbiont’s molecular mechanisms to persist inside the host.

## RESULTS AND DISCUSSION

### Interaction analysis of a chemosynthetic deep-sea symbiosis.

**FIG 1:**
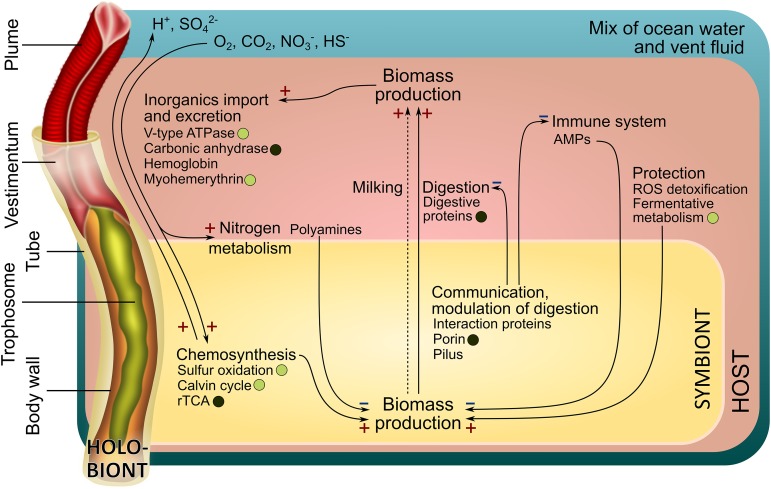
Main interactions in the *Riftia* symbiosis. "HOST" refers to processes in *Riftia* host tissues, while "SYMBIONT" refers to processes in the bacterial endosymbiont. A plus sign indicates presumably stimulating interactions, and a minus sign indicates presumably inhibiting interactions. For example, host efforts that protect the symbiont population from oxidative stress, i.e., ROS detoxification and fermentative metabolism (on the right), can promote symbiont biomass production (+). In contrast, host immune system-related proteins and antimicrobial peptides (AMPs) may inhibit symbiont biomass production (−). Circles, where present, indicate that the respective proteins are more abundant in S-rich (energy-rich) specimens (light circles) or S-depleted (energy-limited) specimens (dark circles). The dashed arrow indicates putative transfer of small organic compounds "Milking"; see Text S1, section 3).

We sequenced the *Riftia* host transcriptome *de novo* and combined it with three existing symbiont genomes to create a comprehensive holobiont database for identification of *Riftia* host and symbiont proteins (see Materials and Methods). Our metaproteomic analysis included comparisons between symbiont-containing and symbiont-free tissues of specimens with light and dark trophosomes. As trophosome color and bacterial sulfur content are directly correlated (27, 28), samples from specimens with light and dark trophosomes will here be referred to as sulfur-rich (S-rich) and sulfur-depleted (S-depleted) samples, respectively. A fully replicated data set and stringent study design enabled us to find statistically significant differences in individual protein abundance between sample types as well as abundance differences between functional protein groups. For an overview of all identified proteins, see Text S1, section 1, and Fig. S1 in the supplemental material. We identified numerous molecular interaction processes (Fig. 1), including (i) metabolite exchange between host and symbiont, (ii) host strategies of symbiont maintenance, and (iii) symbiont mechanisms to persist inside the host. Furthermore, we found that (iv) sulfur availability affects symbiotic interactions in *Riftia*. Beyond the results presented here, our comprehensive metaproteome data sets and our newly established transcriptome-based *Riftia* host database (all available from the PRIDE archive; see below) also provide a valuable resource for future *Riftia* studies and microbe-eukaryote symbiosis research in general.

10.1128/mBio.02243-19.1 Text S1 Supplementary Results and Discussion with supplemental figures. Download Text S1, PDF file, 1.9 MB. Copyright © 2019 Hinzke et al. 2019 Hinzke et al. This content is distributed under the terms of the Creative Commons Attribution 4.0 International license.

### Metabolite exchange between host and symbiont. (i) *Riftia* digests its symbionts for nutrition.

**FIG 2:**
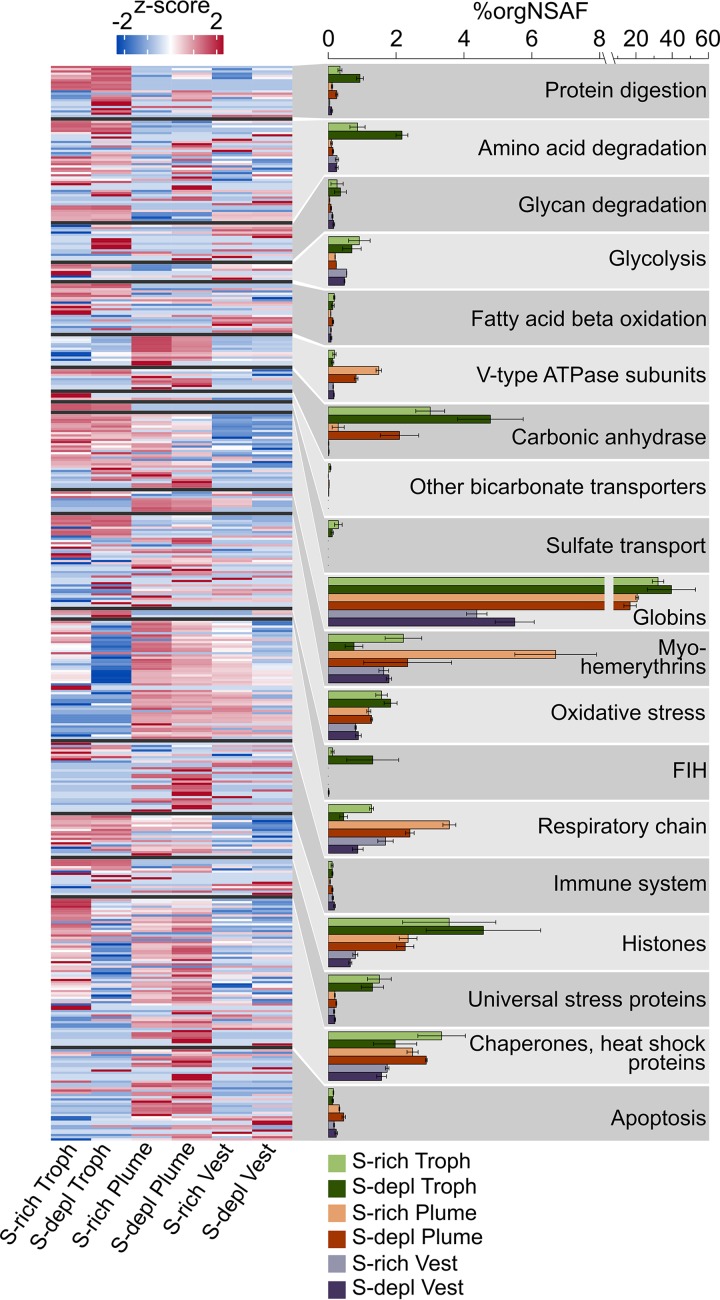
Functional groups of selected *Riftia* host proteins and their relative abundances in tissue samples. The heatmap shows log-normalized, centered, and scaled protein abundances. The bar chart shows summed abundances in %orgNSAF (percent normalized spectral abundance factor per organism, i.e., of all host proteins) of all proteins in the respective category. Error bars indicate standard error of the mean. Note the different scaling in the right part of the *x* axis. The “Chaperones, heat shock proteins” category also includes chaperonins and Clp proteases. FIH, factor inhibiting hypoxia-inducible factor 1α. S-depl, S depleted. Vest, vestimentum. Troph, trophosome. For a list of all identified proteins and their abundances, see Table S1a. (Categories presented in this figure are labeled with X in Table S1a in the column labeled Figure 2. The table can be filtered for these categories.)

Our results suggest that the main mode of nutrient transfer from symbiont to host is the active digestion of symbiont cells, and that this process might involve endosome-like maturation of symbiont-containing vesicles. We detected a total of 113 host enzymes involved in protein, amino acid, and glycan degradation, as well as in glycolysis and fatty acid beta oxidation. Twenty-two of these proteins were significantly more abundant in trophosome samples than in the other tissues (Table 1). Overall, nearly all of the respective protein groups had higher abundances (i.e., higher organism-specific normalized spectral abundance factor values, or %orgNSAF) in the symbiont-bearing trophosome than in other tissues, both in S-rich and S-depleted specimens (Fig. 2). Many of the protein degradation-related proteins contain signal peptides and thus are likely either contained in lysosomes or secreted into the symbiont-containing vesicles to digest the symbiont cells (Table 1 and Table S1a).

**TABLE 1:** Proteins which are putatively involved in symbiont digestion and which had significantly higher abundances in trophosome samples than in other tissues of S-rich and S-depleted specimens

Accession	Description	Sig in^*a*^:		Secreted/membrane^*b*^
		S-rich troph	S-depl troph	
Protein digestion				
Host_DN32373_c0_g1_i1::g.193014	Cathepsin Z	x	x	M
Host_DN34261_c0_g1_i1::g.35886	Cathepsin B	x	x	S
Host_DN38047_c1_g1_i1::g.177385	Cathepsin Z	x	x	M
Host_DN41150_c0_g1_i1::g.101468	Cathepsin L1	x	x	S
Host_DN34118_c0_g1_i3::g.155432	Digestive cysteine proteinase 2	x	x	S
Host_DN39514_c3_g1_i1::g.201492	Legumain	x	x	S
Host_DN34848_c0_g1_i1::g.215091	Dipeptidyl peptidase 1	o	x	S
Amino acid degradation				
Host_DN37934_c0_g3_i4::g.212722	4-Hydroxyphenylpyruvate dioxygenase	x	x	S
Host_DN35553_c0_g1_i1::g.72896	Maleylacetoacetate isomerase	x	x	
Host_DN37934_c0_g3_i6::g.212725	4-Hydroxyphenylpyruvate dioxygenase	x	x	
Host_DN40417_c0_g1_i7::g.93374	d-Aspartate oxidase	x	x	Possibly M
Host_DN41135_c1_g1_i1::g.101501	Homogentisate 1,2-dioxygenase	x	x	
Host_DN39303_c6_g1_i3::g.66273	Urocanate hydratase	x	x	
Host_DN37934_c0_g3_i11::g.212729	4-Hydroxyphenylpyruvate dioxygenase	o	x	
Host_DN39293_c0_g3_i16::g.11113	Histidine ammonia-lyase	o	x	
Host_DN41135_c1_g1_i2::g.101503	Homogentisate 1,2-dioxygenase	o	x	
Host_DN40306_c1_g4_i8::g.129962	Aminoacylase-1	o	x	
Glycan degradation				
Host_DN36692_c1_g2_i4::g.169924	Lysosomal alpha-glucosidase	x	x	M/possibly S
Host_DN36692_c1_g2_i3::g.169923	Glucoamylase 1	o	x	
Host_DN37016_c0_g1_i1::g.156600	Lysosomal alpha-mannosidase	o	x	S
Fatty acid beta oxidation				
Host_DN34874_c0_g1_i9::g.215370	Propionyl-coenzyme A carboxylase beta chain, mitochondrial	x	o	
Host_DN41664_c1_g5_i6::g.166806	Peroxisomal bifunctional enzyme	o	x	

a Sig, Significance (x, significant; o, nonsignificant; false discovery rate, 0.05); troph, trophosome; S-depl, S depleted.

b Subcellular localization (M, membrane-associated; S, secreted) was predicted using Phobius, TMHMM, and SignalP. Possibly M or S indicates localization prediction based on one tool only.

10.1128/mBio.02243-19.2 Table S1 (a) All *Riftia* host proteins identified in this study. (b) Potential autophagy-related *Riftia* proteins. (c) Potential *Riftia* antimicrobial peptides. (d) Genomes and metagenomes used for SMART analysis of eukaryote-like protein structures. (e) Sampling dates, cruise number, and number of biological replicates of *Riftia* samples used in this study. (f) Number of proteins with significant abundance differences in pairwise comparisons of *Riftia* tissues. (g) All *Riftia* symbiont proteins identified in this study. (h) Hemerythrin and myohemerythrin isoforms in *Riftia* and other invertebrates used for alignment in Text S1, Fig. S3. (i) Carbonic anhydrase isoforms in *Riftia* as detected in this study and described in the literature. Download Table S1, XLSX file, 1.8 MB. Copyright © 2019 Hinzke et al. 2019 Hinzke et al. This content is distributed under the terms of the Creative Commons Attribution 4.0 International license.

Our findings are in accordance with previous biochemical, autoradiographic, and microscopic studies, which suggested symbiont digestion in the *Riftia* trophosome (14, 36–38). Moreover, abundant degradative enzymes and symbiont digestion appear to be common in other mutualistic symbioses as well, including deep-sea mussels (39, 40), shallow-water clams (41, 42), and the gutless oligochaete Olavius algarvensis (43, 44).

**FIG 3:**
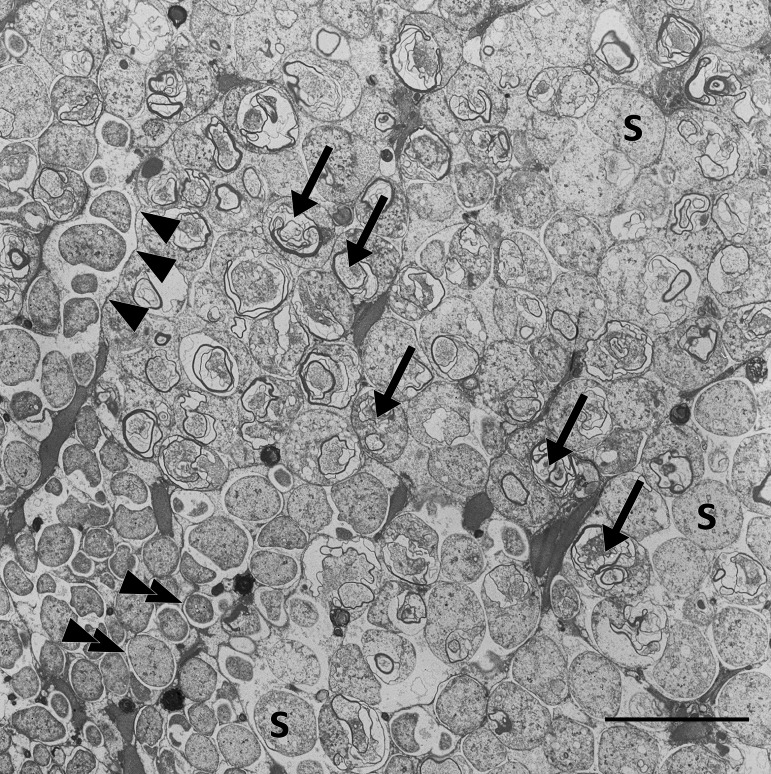
Transmission electron micrograph of a *Riftia* trophosome tissue section. Within the lobular trophosome tissue, this section shows the median and peripheral zones of an individual lobule with host bacteriocytes containing intracellular coccoid symbionts (S) located in dedicated vesicles (arrowheads, bacteriocyte membrane; double arrowheads, vesicle membrane). While the lower left area of the image shows mostly intact symbiont cells, arrows in the central area point to symbiont cells in the state of digestion by the host, where cell degradation is indicated by the presence of lamellar bodies. Image brightness and contrast were adjusted for visual clarity. Scale bar, 10 μm.

Our metaproteome analysis suggests that symbiont digestion in *Riftia* involves maturation of symbiont-containing host vesicles in a process resembling the maturation of endosomes. Endosomes form after endocytosis of extracellular compounds and mature from early to late endosomes, which ultimately fuse with lysosomes (45). The endosome-associated proteins Rab5 and Rab7 showed significantly higher abundances in trophosome samples than in other host tissues (Table S1a). Rab5 and Rab7 localize to early and late stages, respectively, of endosomes and autophagosomes and are markers for these recycling-related organelles (45–47). The idea of symbiont degradation via an endosome-like maturation process in *Riftia* is additionally supported by our transmission electron microscopy (TEM) images of *Riftia* bacteriocytes (Fig. 3), which showed multilamellar bodies. These myelin-like structures can form in endosomes (48) and also during autophagic digestion and have, therefore, previously been attributed to autophagy in the *Riftia* trophosome (37). However, our results suggest that autophagy plays a less prominent role in symbiont digestion, as we detected only two autophagy-related proteins (Table S1b) in the trophosome metaproteome.

Moreover, only 12 of 41 detected apoptosis-related *Riftia* proteins were identified in the trophosome, mostly with similar or significantly lower abundances than in other tissues, and caspases, the main apoptotic effectors, were not detected at all on the protein level in trophosome samples (see also Text S1, section 2). These results suggest that bacteriocyte cell death, which follows after symbiont digestion, probably does not involve apoptosis. This contradicts previous observations (37) but is in line with microscopic results, which did not indicate apoptosis in the trophosome (49). We therefore suggest that an alternative, nonapoptotic cell death mechanism exists in *Riftia* trophosomes. A nonapoptotic, nonautophagic cell death mechanism was recently described in pea aphid bacteriocytes (50). In the aphids, the proposed mechanism involved hypervacuolation of host bacteriocytes, which, however, was not observed in the *Riftia* trophosome. Also, in cancer cells, a caspase-independent (nonapoptotic) cell death mechanism was described, which involves the lysosomal protease cathepsin B (51), a putative regulator of lysosome production and autophagic activity (52). As cathepsin B was significantly more abundant in trophosome than in other *Riftia* tissues, we speculate that this protease, among other degradative enzymes, is involved in controlled cell death in the *Riftia* trophosome.

Besides symbiont digestion, a second mode of nutrient transfer, the release of small organic carbon compounds by intact symbionts (termed “milking”), was suggested to be present in *Riftia* (36, 53). Our calculated δ^13^C ratios might support this hypothesis (Text S1, section 3). However, as we did not detect dedicated symbiont exporters for organic acids or sugars on the proteome level, nutrient transfer by milking is probably less relevant for overall host nutrition than symbiont digestion.

### (ii) *Riftia* dedicates a substantial part of its proteome to provisioning the symbionts with O_2_, sulfide, and CO_2_.

We found highly abundant and diverse globins, myohemerythrins, V-type ATPase subunits, and carbonic anhydrases in the host proteome (Fig. 2), indicating that *Riftia* dedicates a substantial part of its proteome to provisioning the symbiont with all necessary substrates for chemosynthesis.

Globins made up about one-third of all trophosomal host proteins and one-fifth of the total proteome in the plume (i.e., the worm’s symbiont-free gas exchange organ; Fig. 2), with extracellular hemoglobins being particularly abundant (in sum, 32 to 40 %orgNSAF in trophosome and 17 to 21% in plume samples). *Riftia* has three distinct extracellular hemoglobins composed of globin chains and, in the case of the hexagonal bilayer hemoglobin, globin linker chains (54–56). We detected several of these subunits, including isoforms that are (to our knowledge) hitherto undescribed (Table S1a). *Riftia’s* extracellular hemoglobins have been shown to bind both O_2_ and sulfide (56, 57; reviewed in references 58 and 59). Consequently, abundant hemoglobins in the highly vascularized plume would ensure efficient uptake of these compounds for transport to the symbionts (see Text S1, section 4, for more details on sulfur metabolism in the host). Moreover, reversible O_2_ and sulfide binding to abundant hemoglobins in the trophosome not only provides the bacteria with chemosynthetic substrates and prevents spontaneous sulfide oxidation but also protects the symbionts from oxygen (60). As suggested previously (23), *Riftia* symbionts are microaerophilic, i.e., sensitive to high oxygen levels. This idea is corroborated by the presence of several ROS-scavenging enzymes (superoxide dismutase, alkyl hydroperoxide reductase, and rubrerythrin) and cytochrome *c* oxidase *cbb*_3_ subunits in the symbiont metaproteome. *cbb*_3_ has a high affinity for oxygen and participates in microaerobic respiration (61). In addition to extracellular hemoglobins, we identified four low-abundance (0.002 to 0.084 %orgNSAF) globins that are probably intracellular and might store O_2_ (Text S1, section 5).

Besides hemoglobins, myohemerythrins were detected in all tissues, with particularly high abundances of 6.7 %orgNSAF in S-rich plumes. With their comparatively high oxygen-binding capacity (62), hemerythrins could facilitate oxygen uptake from the environment into the plume and are possibly also involved in O_2_ storage and intracellular transport in *Riftia*. Moreover, the abundance distribution of the nine detected myohemerythrins suggests a tissue-specific function (Text S1, section 6).

V-type ATPase subunits were found with highest total abundances of up to 1.5 %orgNSAF in *Riftia* plumes (Fig. 2), and almost all of the detected subunits were significantly more abundant or exclusively detected in the plumes. V-type ATPases have a pivotal function in regulating internal pH and CO_2_ uptake (63) and thus in symbiont provisioning. The high energy demand of V-type ATPase-dependent pH regulation could be met via a relatively higher respiration activity in the plume, as indicated by comparatively higher total abundances of respiratory chain proteins (Fig. 2), ATP synthase, and mitochondrial ribosomal proteins in this tissue. Additionally, carbonic anhydrase (CA), another important enzyme for CO_2_ uptake, was detected in all tissues. While we observed tissue-specific abundance patterns of individual CAs (Text S1, section 7 and Fig. S4), overall CA abundance was highest in the trophosome (Fig. 2). CA facilitates CO_2_ diffusion into the plume by converting it to HCO_3_^−^ (63, 64) and likely back-converts the HCO_3_^−^ to CO_2_ for fixation by the symbionts in the trophosome. Our analysis suggests that three of the *Riftia* CAs are membrane bound (Text S1, section 7) and could thus facilitate CO_2_ diffusion into bacteriocytes by converting HCO_3_^−^ to CO_2_ on the bacteriocyte cell surface (65, 66). Transport of HCO_3_^−^ to the bacteriocytes could be mediated by sodium bicarbonate exchangers, which we identified in trophosome and plume samples (Table S1a).

While carbon for fixation by the *Riftia* symbiont is likely mainly transported in the form of CO_2_/HCO_3_^−^, the host may additionally pre-fix CO_2_ into organic C_4_ compounds, which are then transported to the symbiont (67). We did identify host phosphoenolpyruvate carboxykinase and pyruvate carboxylase, which could be involved in this process (Text S1, section 8).

### (iii) *Riftia’s* nitrogen metabolism depends less on the symbiont than previously assumed.

**FIG 4:**
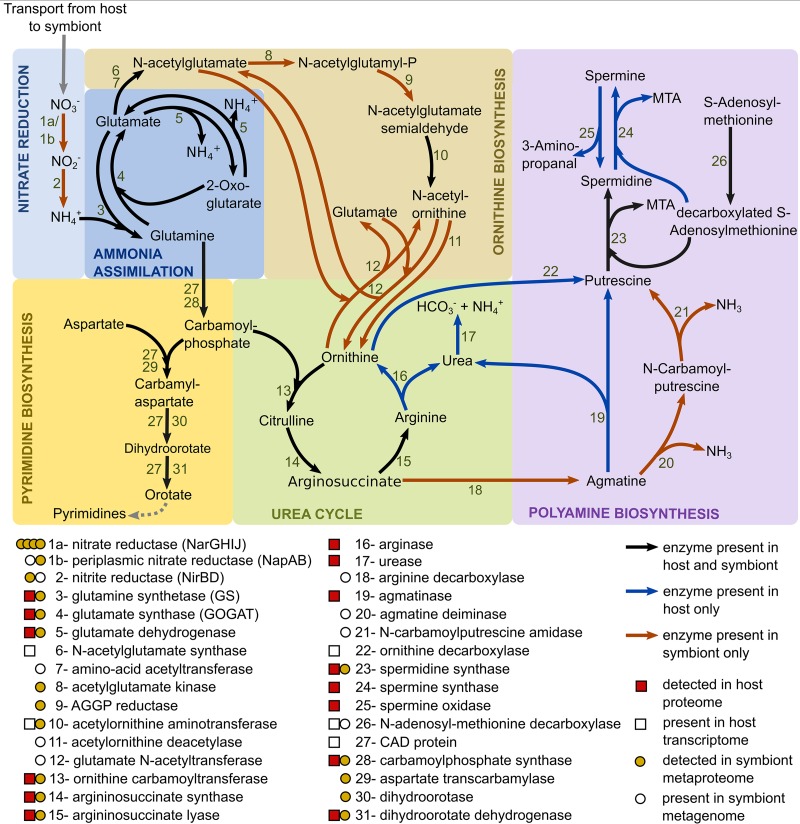
Main nitrogen metabolic pathways in *Riftia* symbiosis. AGGP reductase, *N*-acetyl-gamma-glutamyl-phosphate reductase; CAD protein, multifunctional carbamoyl-phosphate synthetase 2, aspartate transcarbamoylase, and dihydroorotase protein; MTA, 5′-methylthioadenosine. Note that the symbiont might also be capable of nitrate respiration (25, 60), which is not depicted here.

*Riftia* symbionts supply their host not only with carbon and energy sources but likely also with ammonium produced by bacterial nitrate reduction (Fig. 4 and Text S1, section 9). However, with regard to the subsequent metabolization of organic nitrogen, the host might be more self-sufficient than previously thought: previous biochemical analyses suggested that only the symbiont, but not the host, can *de novo* synthesize pyrimidines (68) and produce polyamines (69). In contrast to those studies, we found the multifunctional CAD protein (which combines the three enzyme functions carbamoyl-phosphate synthetase 2, aspartate transcarbamoylase, and dihydroorotase) in the *Riftia* host metatranscriptome, suggesting that the host can catalyze the first steps of pyrimidine synthesis. As we did not detect CAD protein on the protein level, expression levels and associated activities in the host are likely rather low, and most of the pyrimidine demand could be satisfied by digesting symbionts. In addition, we found key genes involved in polyamine synthesis in the host's metatranscriptome and also detected several of the respective proteins in the host's metaproteome (Fig. 4). Our results suggest that, while both *Riftia* symbiosis partners can synthesize spermidine, in fact only the host is able to generate spermine. Host spermidine synthase and spermine synthase were exclusively detected in trophosome samples in our study, suggesting that the polyamines produced by these proteins have a role in symbiont-host interactions. They could, for example, be involved in restricting the symbiont to its cell compartment, i.e., the bacteriocyte vesicle, as suggested for bacterial pathogens (Text S1, section 10). In addition, only the host seems to possess a full urea cycle and might degrade not only its own but also nitrogen-containing metabolites of the symbiont (Text S1, section 9). These results suggest that the symbiont provides the host with necessary metabolic energy and building blocks for biosynthesis but that the host has also retained key biosynthetic capacities for N-containing organic compounds.

### Host strategies of symbiont maintenance.

**(i) *Riftia* protects its symbiont from oxidative damage and may even generate hypoxic conditions in the trophosome.** We found several reactive oxygen species (ROS)-scavenging enzymes (superoxide dismutase, peroxiredoxin, and glutathione *S*-transferase) as well as proteins indicative of anaerobic metabolism and universal stress proteins with significantly higher individual abundance and in higher total amounts (summed %orgNSAF) in the trophosome than in other tissues (Fig. 2 and Text S1, section 11). *Riftia*’s ROS-detoxifying enzymes probably protect not only the host but also the microaerophilic symbiont against ROS. Upregulation of host proteins involved in ROS detoxification was previously shown in the *Wolbachia* symbiosis (70, 71). Additionally, malate dehydrogenase was highly abundant in trophosomes. This enzyme is regularly observed in different invertebrates under anaerobic conditions (72) and is involved in maintaining redox balance during anaerobiosis (73). Therefore, the host might generate hypoxic conditions in the trophosome, as also indicated by the overall lower abundance of host respiratory chain proteins in trophosome than in other tissues of both S-rich and S-depleted specimens. We also detected hypoxia-inducible factor 1-alpha inhibitors (factor inhibiting HIF1a; FIH) almost exclusively in trophosome samples, which further supports the idea that free oxygen concentrations in the trophosome are low. This is in line with the high oxygen-binding capacity of *Riftia* hemoglobins (23, 60) and with the suggestion of fermentative metabolism under hypoxic and even normoxic conditions in *Riftia* based on biochemical results (74). Taken together, lower oxygen concentration in the trophosome, (partial) anaerobic host metabolism, and host ROS-detoxifying enzymes in this tissue would not only protect the symbionts from oxidative damage but also decrease the competition between the *Riftia* host and its symbionts for oxygen.

**(ii) The *Riftia* immune system might be involved in symbiont population control.** We detected several proteins that potentially are involved in a specific immune reaction of *Riftia* against its symbiont in the trophosome. Two bactericidal permeability-increasing proteins (BPIPs) were detected, one exclusively in the trophosome, the other only in the plume. BPIPs act specifically against Gram-negative bacteria, causing initial growth arrest and subsequent killing due to inner membrane damage (75). In *Riftia*, BPIPs could be involved in keeping the symbiont population under control, e.g., as part of the digestion process or by preventing the symbionts from leaving their intracellular host vesicles. Likewise, in the *Vibrio*-squid symbiosis, BPIPs have been implied in restricting the symbiont population to the light organ (76). In addition to BPIPs, a pathogen-related protein (PRP) was present in all replicates of S-rich trophosome but absent from all other tissues. In plants, PRPs accumulate during defense responses against pathogens (reviewed in reference 77). PRPs have also been described in nematodes (78) and humans (79), although their function remains elusive.

We also found that histones had overall higher abundance in *Riftia* trophosome than in other tissues. Four of these histones were significantly more abundant in trophosomes than in other tissues, and three additional histones were exclusively detected in trophosome samples (Table S1a). Besides being crucial for DNA interactions, histones and histone-derived peptides can have antimicrobial effects (80–82). A BLASTP search of the detected *Riftia* histones against the antimicrobial peptide (AMP) database APD3 (83) gave hits for four of the *Riftia* histones (Table S1c), stimulating the speculation that these histones have antimicrobial properties. While AMP-like histone-derived peptides in the plume might be involved in defense against environmental microbes, the high abundance of histones in the trophosome could point to a function in host-symbiont interaction. Host-derived AMPs could, for example, be involved in controlling the symbiont’s cell cycle. In their life cycle, the symbionts apparently differentiate from actively dividing stem cells into growing but nondividing larger cells (49). As various AMPs were shown to inhibit cell division or septum formation and to cause filamentous cell morphologies (reviewed in reference 84), we speculate that *Riftia* AMPs inhibit cell division as well, e.g., via interaction with the symbiont protein GroEL. Interaction between a host AMP and a symbiont GroEL has been proposed to lead to cell elongation of bacterial weevil symbionts (85). A role of histones and histone-derived peptides in immune system responses has been described or suggested in various other organisms, including catfish (80), Komodo dragons (86), toads (81), and humans (82).

Beyond these and a few other individual immunity-related proteins, we did not observe an overall higher abundance of host immune system proteins (such as lysozyme, complement system proteins, or peptidoglycan recognition proteins) in the trophosome than in symbiont-free tissues. This indicates that the host immune system does not play a major role in controlling symbiont population size. More likely, symbiont population control might to a large part be a result of digestion of symbionts (a “mowing” process), which effectively prevents the symbionts from escaping their compartments and/or overgrowing the host. Nevertheless, the immune system might be involved in phage protection and symbiont recognition during establishment of the symbiosis (Text S1, section 12).

### Symbiont persistence mechanisms. (i) Eukaryote-like protein structures in the symbiont might be involved in host communication.

**FIG 5:**
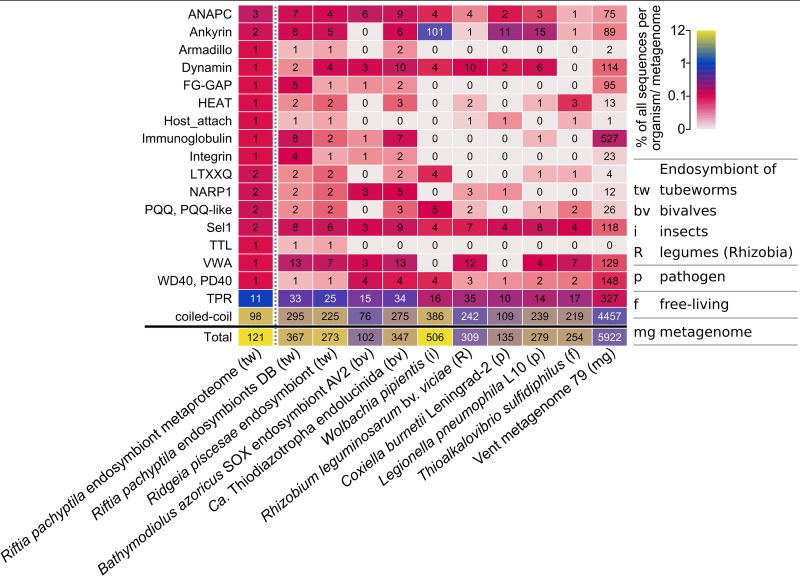
Selected domains with eukaryote-like structures and with putative functions in symbiont-host interactions in the *Riftia* symbiont and in selected other organisms and metagenomes. Color scale shows the percentage of genes/proteins containing the respective domain relative to all gene/protein sequences in this organism or metagenome. Numbers indicate the total number of genes/proteins containing the respective domain. For an overview of all analyzed organisms and domains, see Text S1, Fig. S5. For details on the organisms and communities, see Table S1d. The vent metagenome was sampled from hydrothermal vent fluid at a diffuse-flow vent site (Crab Spa) (137), which also houses *Riftia*. For further information about the selected protein groups, see Table S2. *Riftia pachyptila* endosymbiont metaproteome refers to the *Riftia* symbiont proteins detected in this study.

The metagenome of the *Riftia* symbiont “*Ca.* E. persephone” encodes several protein groups with possible roles in symbiont-host interactions, including eukaryote-like protein (ELP) structures, as revealed by our SMART analysis (Table S2). We detected more than 100 of these symbiont proteins in the trophosome samples (Fig. 5), which points to a symbiosis-relevant function.

10.1128/mBio.02243-19.3 TABLE S2 Domains and protein families with a putative role in host-symbiont interactions. Download Table S2, PDF file, 0.4 MB. Copyright © 2019 Hinzke et al. 2019 Hinzke et al. This content is distributed under the terms of the Creative Commons Attribution 4.0 International license.

Among the ELPs detected in the symbiont metaproteome were two ankyrin repeat-containing proteins, which contain a signal peptide and are therefore likely secreted (predicted by Phobius, http://phobius.sbc.su.se/). Ankyrin repeats were found to mediate protein-protein interactions (87). In the sponge *Cymbastela concentrica*, symbiont ankyrins were proposed to interact with the eukaryote’s phagocytosis system: the symbiont ankyrins were heterologously expressed in Escherichia coli and led to inhibition of phagocytosis by amoebae (88). Likewise, a secreted Legionella pneumophila ankyrin protein apparently interferes with host endosome maturation (89). The “*Ca.* E. persephone” ankyrin repeat-containing proteins therefore could directly interact with host proteins as well, e.g., to modulate endosome maturation and thus to interfere with symbiont digestion by the host. Similarly, proteins with tetratricopeptide repeat (TPR)/Sel1 domains, which we also detected in the “*Ca.* E. persephone” metaproteome, have been shown to impact phagocytosis by amoebae (90).

The *Riftia* symbiont furthermore encodes eukaryote-like proteins of the tubulin-tyrosine ligase family (TTL proteins). These proteins posttranslationally modify tubulin and thus interact with the eukaryotic cytoskeleton (91). We found one TTL protein in the “*Ca.* E. persephone” metaproteome. Other protein groups that are involved in protein-protein interactions in eukaryotes, e.g., with cytoskeletal proteins, and that we detected in “*Ca.* E. persephone” include armadillo repeat proteins (92) and HEAT repeat-containing proteins (93). As several of the protein structures analyzed here are also found in other mutualistic symbionts and pathogens (Text S1, section 13, and Table S2), it is conceivable that parallels exist between interaction processes of mutualistic and pathogenic associations and that the *Riftia* symbiont employs a strategy similar to that of pathogens to communicate with its host on the molecular level.

### (ii) Symbiont membrane proteins may export effector proteins into host cells and lead to strain adaptation.

We detected various outer membrane-related proteins in the “*Ca.* E. persephone” proteome, including a porin (Sym_EGV52132.1), which was one of the most abundantly expressed symbiont proteins, and 12 type IV pilus (T4P) system proteins (PilQ, PilF, PilC, PilBTU, PilM, PilN, PilP, FimV, PilH, and PilY1). Five additional T4P structure proteins were encoded in the metagenome (*pilVWXE* and *pilO*). These proteins are in direct contact with the host cells and thus are likely involved in interactions between both symbiosis partners, including such processes that facilitate the symbiont’s persistence inside the host cells.

The abundant symbiont porins could transport effector molecules, e.g., to modulate digestion by the host. A role of porins in effector transport during symbiosis has been hypothesized for Vibrio fischeri OmpU, a channel protein that is important for symbiont recognition by the squid host (94).

The T4P system is a complex structure, which in Pseudomonas aeruginosa comprises more than 40 proteins, including structural and regulatory proteins (95). It can have several functions in different species: adhesion, secretion, and natural transformation (95–98). As the “*Ca.* E. persephone” T4P system likely is not involved in adhesion to host cells during symbiosis (although it might be during the initial infection), it could participate in protein secretion and/or natural transformation. The *Riftia* symbiont’s T4P system could export putative effector proteins (e.g., ankyrins and SET domain proteins; Text S1, sections 13 and 14) for host interactions. Interestingly, in the pathogen Francisella tularensis subsp. *novicida*, a T4P structure is involved in secretion of infection-moderating proteins (97).

Besides their putative function in effector protein export, symbiont membrane proteins may also lead to bacterial strain adaptation. The *Riftia* symbiont population is polyclonal, i.e., although there is only one 16S rRNA phylotype, this phylotype consists of several distinct strains (20). T4P system-mediated exchange of genetic material between different symbiont strains would add to this diversity in the symbiosis and might additionally enable exchange of symbiosis-related genes within the free-living “*Ca.* E. persephone” population. Natural transformation in symbionts has only recently been shown for V. fischeri in culture (99) and the earthworm symbiont Verminephrobacter eiseniae, which likely employs a T4P structure for DNA uptake (98). As microbial cell densities are comparatively high in eukaryote-prokaryote mutualisms, natural transformation in these systems might actually be more common than previously recognized. While mostly only one to three symbiont cells are located in one host vesicle, individual vesicles with up to 14 symbiont cells have also been reported (49), which might allow for exchange of genetic material. The proposed DNA uptake by the *Riftia* symbiont may not only facilitate exchange between symbiont strains but also promote horizontal gene transfer between host and symbiont, e.g., of eukaryote-like proteins. This hypothesis, as well as the speculation that “*Ca.* E. persephone” is capable of conjugation (Text S1, section 14), certainly warrant further investigations.

### S availability affects symbiotic interactions in *Riftia*. (i) S-depleted *Riftia* hosts digest more symbionts than S-rich specimens.

**FIG 6:**
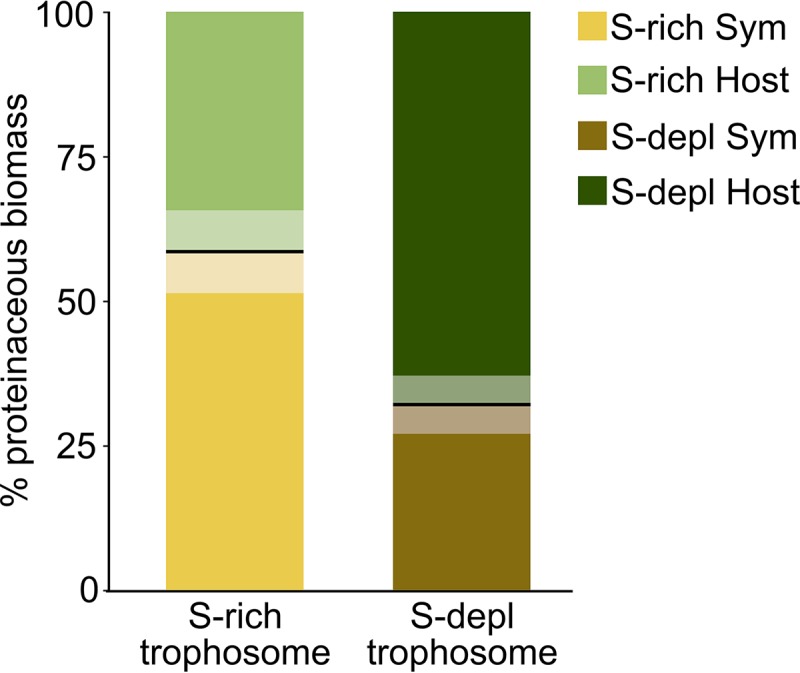
Percent proteinaceous biomass contributions of host and symbiont as calculated from the share of host and symbiont spectral counts in all spectral counts of the respective samples (127; see Materials and Methods for details). Boldface lines indicate the means, and semitransparent areas indicate standard error of the mean. Sym, symbiont; S-depl, S depleted.

We compared the metaproteomes of *Riftia* specimens with and without stored sulfur (i.e., energy-rich versus energy-depleted specimens; Text S1, Fig. S1) to examine how energy availability impacts symbiotic interactions (see Table S1f for total numbers of differentially abundant proteins). Metabolite transfer is apparently especially influenced by the energy regime: the host supposedly relies more on symbiont digestion in times of S shortage. Proteinaceous symbiont biomass was notably lower in S-depleted trophosomes (32%) than in S-rich trophosomes (58%) (Fig. 6). Simultaneously, overall abundances for several groups of host digestive enzymes were higher in S-starved trophosomes (Fig. 2), and a number of individual host proteins were significantly more abundant in these S-depleted samples, such as enzymes involved in protein digestion (including cathepsin B), amino acid degradation, the late endosome-related protein Rab7, and histones (Table S1a). One reason for this supposed increase in symbiont digestion in S-depleted trophosomes could be a lower nutritional value of the energy-depleted symbionts. S-depleted symbionts have lower abundances of enzymes involved in sulfur oxidation, probably due to lower S availability. Therefore, less energy might be available for biosynthesis under S depletion, rendering the symbiont less nutritious for the host. The animal would then, especially if S depletion is prolonged, have to rely on increased symbiont digestion in order to still satisfy its basal metabolic demands. Thus, S-depleted hosts may, despite increased symbiont digestion, have less energy available. This idea is supported by the observation that host proteins involved in the energy-generating glycolysis, tricarboxylic acid (TCA) cycle, respiratory chain, ATP synthesis, and biosynthetic pathways were less abundant in S-depleted trophosomes than in S-rich trophosomes. Concomitant with the postulated lower nutritional value of S-depleted symbionts, the Calvin cycle key enzyme RubisCO had an about 10-fold lower abundance in S-depleted symbionts. Abundance of the reverse TCA (rTCA) cycle key enzyme ATP citrate lyase (NCBI accession no. EGV51152.1), on the other hand, was slightly higher in S-depleted symbionts than in S-rich symbionts, albeit only 1.4-fold. Under S-depleted conditions, symbionts apparently rely relatively more on the rTCA cycle, which is more energy efficient than the Calvin cycle (35). The Calvin cycle could be used in addition to the rTCA cycle under favorable conditions to maximize carbon fixation. Moreover, symbiont enzymes involved in translation were overall more abundant in S-rich trophosomes than in S-depleted trophosomes. Less protein biosynthesis in S-depleted symbionts would not only impact the nutritional value of these symbionts but also directly decrease the proteinaceous symbiont biomass. The reason for the lower proteinaceous biomass of symbionts in S-depleted trophosomes is, therefore, probably 2-fold: the host digests more symbionts and the symbionts produce less biomass than in energy-rich trophosomes.

These findings are in contrast to previous results (29), which showed no significant differences in autotrophic activity and symbiont abundance between *Riftia* specimens from high- versus low-sulfide habitats. Increased symbiont digestion may be a short-term adaptation to fluctuating environmental conditions, whereas under long-term low-S conditions the symbiosis might adapt by other means, e.g., by reduced growth rates. Decrease in symbiont abundance or total protein under energy-limiting conditions also has been noted in *Bathymodiolus* (100) and Codakia orbicularis bivalves (42) as well as in *O. algarvensis* oligochaetes (43). Thus, relying on the symbionts as a nutrient source also under unfavorable conditions appears to be a common symbiosis mechanism that would ensure survival of the host and a subset of the symbiont population, ultimately prolonging survival of the individual holobiont.

### (ii) S availability influences CO_2_ uptake, pH regulation, and O_2_ regime in the *Riftia* host.

S-depleted hosts seem to invest relatively more biosynthetic capacities in CO_2_ uptake and less in pH regulation, and their trophosomes are supposedly less hypoxic than those of S-rich hosts (Text S1, sections 11 and 15). At the same time, S availability appeared to have little influence on non-symbiont-related processes in the host, as only very few (i.e., <10) individual proteins differed significantly in abundance between S-rich and S-depleted plume and vestimentum samples. This indicates that the host’s metabolism is very well buffered against changes in environmental conditions.

### (iii) Higher digestion pressure might result in symbiont countermeasures.

A putative “*Ca.* E. persephone” dodecin was significantly more abundant in S-depleted *Riftia* specimens than in S-rich specimens. This protein might be involved in protecting the symbiont against oxygen and/or digestion stress (Text S1, section 14). A symbiont porin, which was also significantly more abundant in S-depleted specimens, might be involved in counteracting the supposedly higher digestion pressure (described above) (Text S1, section 14).

### Conclusions.

To fully understand the biology of organisms, it is crucial to study them together with their symbiotic partners as holobionts (101). Given its low complexity, high specificity, and extreme dependence of the host on the symbiont, the association of *Riftia* and its bacterial partner serves as an excellent system to study mutualistic host-microbe interactions. While *Riftia* lives in a unique and remote environment, many of the interactions we identified, like symbiont digestion by the host, high host investment in substrate transfer to the symbiont, host-directed symbiont population control, and eukaryote-like symbiont proteins that could interact with the host's molecular machinery, seem to be critical in other symbiotic associations as well, including insects, mussels, and oligochaetes. These interactions might therefore represent common principles among evolutionarily diverse mutualistic animal-microbe associations.

Our study provides access to the *Riftia* host transcriptome and protein sequences and thus paves the way for future research on host-microbe interactions in *Riftia* and other systems. Promising research directions include the elucidation of protein functions, e.g., of *Riftia* immune system proteins and symbiont eukaryote-like proteins by heterologous gene expression and biochemical assays in model systems. Moreover, our work stimulates future in-depth studies of the molecular mechanisms involved in recognition of both partners during the initial infection of *Riftia* larvae by free-living symbionts. Putative differences between *Riftia*’s short- and long-term adaptation strategies in response to changing environmental conditions also warrant further investigation.

## MATERIALS AND METHODS

### Sampling.

*Riftia* samples were obtained during several research cruises in 2008, 2014, and 2017, with RV *Atlantis*, to the deep-sea hydrothermal vent fields on the East Pacific Rise at 9°50′N, 104°17′W. *Riftia* specimens were collected by the human occupied vehicle *Alvin* or the remotely operated vehicle *Jason* in approximately 2,500-m water depth. Specimens were kept at 4°C in cold seawater until dissection, which was performed within 4 h after recovery. Only healthy-looking specimens were used. Sampling dates for all *Riftia* tissue samples for proteomics, transcriptomics, and transmission electron microscopy (TEM) are summarized in Table S1e in the supplemental material. Different specimens were used for proteomics, transcriptomics, and TEM. *Riftia* specimens were dissected onboard, and samples from four different organs (here referred to as tissues) were stored at –80°C: the lamellae of the tentacular crown were shaved off to provide plume samples, trophosome samples were dissected from whole trophosome, body wall samples were retrieved and washed after removal of the trophosome, and vestimental samples were cut off from the lateral portions of the vestimentum. As trophosome color is directly correlated to the tissue’s elemental sulfur content (27, 28), we classified specimens as sulfur rich (S rich), S depleted, and medium S according to their trophosome color (yellow/light green, dark green/black, or medium green, respectively). To ensure comparability of the classifications, the same light source and dissection tray were used for all samples. Sulfur-rich and sulfur-depleted specimens were used for transcriptome sequencing and comparative metaproteomics, while specimens with medium sulfur content were only used for transcriptome sequencing (Table S1e).

### Extraction of whole-tissue RNA.

RNA was extracted from a total of 22 tissue samples from 9 specimens with high, medium, and low trophosome sulfur content (6× trophosome, 6× body wall, 5× plume, 5× vestimentum) (Fig. 1). Tissue samples were homogenized by bead beating with lysing matrix D (MP Biomedicals) in 1 ml TRIzol (Thermo Fisher Scientific; 3 times at 6.5 m/s for 30 s, with 3 min of cooling on ice between steps). After 5 min of acclimatization to room temperature, samples were applied onto QIAShredder columns (Qiagen) and centrifuged (16,000 × *g*, 3 min, 4°C). Afterwards, RNA was isolated from the aqueous flowthrough according to the TRIzol extraction protocol, with the modification that samples were centrifuged for 20 min at 12,000 × *g* and 4°C for phase separation. Ten micrograms of glycogen was added for RNA precipitation. RNA was washed twice with 75% ethanol and purified using the Norgen RNA clean-up and concentration kit according to the manufacturer’s protocol A, including DNA removal with DNase (Qiagen). Quality of extracted RNA was assessed using NanoDrop (Thermo Fisher Scientific) and Bioanalyzer (Agilent) analyses.

### Transcriptome sequencing and assembly. (i) Transcriptome sequencing.

Transcriptome sequencing was performed employing the TruSeq stranded mRNA [poly(A)-based] library protocol (Illumina) on a HiSeq 4000 (Illumina) according to the manufacturer’s guidelines.

### (ii) Transcriptome assembly.

High-throughput paired-end Illumina sequencing resulted in an average of about 26 million reads per end per library (minimum of 16,045,121 reads per end, maximum of 31,318,532 reads per end; 95% confidence interval, 1,673,590). After demultiplexing and quality checking of reads in FastQC v0.11.5 (102), we trimmed low-quality bases and adapters with Trimmomatic v0.32 (103) using the settings ILLUMINACLIP:AllAdapters.fa:2:30:10 SLIDINGWINDOW:4:20 and LEADING:5 TRAILING:5 HEADCROP:15 MINLEN:75. Although bacterial mRNA does not possess a poly(A) tail, previous research has shown that bacterial reads can still be present in poly(A)-enriched RNA-sequencing libraries (104). To filter out potential symbiont contaminations from our host transcriptomes, we used the Bowtie 2 v2.2.9 aligner (105) in very-sensitive mode to map the quality-filtered paired-end reads against the published genomes of the endosymbionts of *Riftia* (Riftia1, NCBI locus tag prefix RIFP1SYM; Riftia2, locus tag prefix RIFP2SYM) and Tevnia jerichonana (34). Unmapped paired-end reads were subsequently extracted using SAMtools v1.4.1 (106). Potential environmental sequence contaminations from sample handling were excluded with DeconSeq v0.4.3 (107), using coverage and identity thresholds of 0.90 and 0.95, respectively. The decontaminated host reads were normalized, pooled, and assembled with Trinity v2.3.2 (108). To optimize the transcriptome assembly, we performed four different assemblies with different parameters and input files: (i) only paired reads, (ii) paired and unpaired reads, (iii) only paired reads plus Jaccard-clip option (to reduce chimeras), and (iv) paired and unpaired reads plus Jaccard-clip option.

To assess the completeness of the different assemblies, we compared our transcriptomes to the BUSCO v2.0 eukaryote and metazoan orthologous data sets (109). Overall, the best results in terms of transcriptome completeness and quality were obtained by the assembly approach using paired and unpaired reads plus the Jaccard-clip option (complete BUSCO, 99.0%) (Table S3). This data set was used for all further analyses.

10.1128/mBio.02243-19.4 TABLE S3 Transcriptome completeness for the four different *Riftia* transcriptome assemblies based on the BUSCO eukaryote and metazoan datasets. Download Table S3, PDF file, 0.3 MB. Copyright © 2019 Hinzke et al. 2019 Hinzke et al. This content is distributed under the terms of the Creative Commons Attribution 4.0 International license.

### (iii) ORF prediction.

TransDecoder v3.0.1 (110) was used to identify coding regions in the assembled transcripts. To improve open reading frame (ORF) prediction, we examined all candidate ORFs for homology to known proteins by searching the Swiss-Prot (http://www.uniprot.org) and Pfam (111) databases (downloaded 3 January 2017) with BLASTP (E value of 1e−05) (112) and HMMER3 (113), respectively. ORFs that were longer than 100 amino acids and/or had a database entry were retained. The FASTA headers of the TransDecoder output files were modified with a custom PERL script to include the BLASTP protein annotations.

### Database generation.

A common database for protein identification of *Riftia* host and symbiont was generated. To this end, host protein sequences were clustered at 95% identity with CD-HIT v. 4.6 (114). For symbiont sequences, the three proteomes of the *Riftia*1 symbiont (NCBI PRJNA60889, ID 60889), *Riftia*2 symbiont (NCBI PRJNA60891, JGI 2600255285), and *Tevnia* symbiont (NCBI PRJNA60887, ID 60887) (34) were used. *Riftia*1 was used as basis for clustering the symbiont protein sequences with CD-Hit-2D (114). Subsequently, the combined symbiont database was clustered at 95% identity. Identifier prefixes were added to distinguish between host and symbiont sequences for Calis-p (115 and see below). Host and symbiont databases were concatenated, and the cRAP database containing common laboratory contaminants (116) was added. The final database contained 71,194 sequences (67,092 host and 3,986 symbiont protein sequences).

### Proteomics sample preparation and analysis.

For metaproteomics analysis, we used three biological replicates per tissue (trophosome, vestimentum, and plume) and condition (specimens with S-rich and S-depleted trophosomes), which resulted in a total of 18 samples. Tissues were disrupted by bead beating for 45 s at 6.0 m/s with lysing matrix D tubes (MP Biomedicals) in SDT buffer (4% [wt/vol] sodium dodecyl sulfate [SDS], 100 mM Tris-HCl, pH 7.6, 0.1 M dithiothreitol [DTT]), followed by heating to 95°C for 10 min. Tryptic peptides were generated following the FASP protocol of Wiśniewski et al. (117), with minor modifications as described by Hamann et al. (118). Peptide concentrations were determined with the Pierce Micro BCA (bicinchoninic acid) assay (Thermo Scientific Pierce) according to the manufacturer’s instructions. The tryptic digest was desalted on-line during liquid chromatography tandem mass spectrometry (LC-MS/MS) analysis.

All samples were analyzed by one-dimensional LC-MS/MS as described by Hinzke et al. (119), using 4-h gradients. Samples were analyzed in a randomized block design (120) and run in technical triplicates. Two technical replicate runs were acquired with a 50-cm analytical column, one with a 75-cm analytical column. To standardize the stable isotope fingerprinting (SIF) analysis (115), human hair was measured in technical duplicate alongside the *Riftia* samples in the replicate run using a 75-cm column.

### Proteomics data evaluation. (i) Protein identification, quantification, and statistical analyses.

For protein identification, MS/MS spectra of combined technical triplicate runs were searched against the combined host and symbiont database using the Sequest HT node in Proteome Discoverer version 2.0.0.802 (Thermo Fisher Scientific) as described in Kleiner et al. (115). For protein abundance estimates, normalized spectral abundance factors (NSAFs) (121) were calculated per sample and organism (%orgNSAF) (122). Statistical evaluation was performed based on spectral counts using the edgeR package (123) in R (124). The edgeR package uses an overdispersed Poisson model for analysis of count data. Overdispersion is moderated across proteins using empirical Bayes methods, and differentially abundant proteins are detected using an overdispersion-adapted analog to Fisher’s exact test (123). We filtered for proteins with at least 10 spectral counts for host proteins and at least 5 spectral counts for symbiont proteins in at least three samples and employed a false discovery rate (FDR) of 0.05 to assign statistical significance to protein abundance differences. For graphical representation, heatmaps were generated with the R package ComplexHeatmaps (125) and intersection plots with the R package UpsetR (126). Protein biomasses of host and symbiont were calculated as described in Kleiner et al. (127). Spectral counts of all symbiont proteins and of all host proteins identified with at least two unique peptides were summed individually, and these two sums were divided by the sum of all spectral counts (host plus symbiont proteins with at least two unique peptides) and multiplied by 100 to give the percentage of proteinaceous biomass for host and symbiont.

δ^13^C values of *Riftia* symbiont and host were calculated from mass spectrometry data with Calis-p (115) using one technical replicate LC-MS/MS run (75-cm analytical column). Human hair was used as a reference material.

### (ii) Protein annotations, functional characterization, and categorization.

Besides the annotations included in the database, proteins where further characterized using the online tools described in Table S4. Proteins were manually categorized into functional groups based on their annotations and on protein function information in the UniProt (128), NCBI (https://www.ncbi.nlm.nih.gov/), and InterPro (129) databases. We used the Transporter Automatic Annotation Pipeline (TransAAP) (http://www.membranetransport.org/transportDB2/TransAAP_login.html) of TransportDB2 (130) and TCDB (131) with gblast 2 (http://www.tcdb.org/labsoftware.php) to annotate transporters in the *Riftia*1 symbiont metagenome database. To detect potential antimicrobial peptides (AMPs) among the host proteins, we searched the detected host proteins against the antimicrobial peptide database APD3 (83) using BLASTP (112) in BLAST+ 2.7.1 (132). Results were filtered for identity of >75% and E value of <0.005. We screened the *Riftia* proteome for homologs of known autophagy-related Drosophila melanogaster proteins (as listed in reference 133) by BLAST searching (BLASTP [112] in BLAST+ 2.8.1 [132]) the *Riftia* host proteome against the respective *Drosophila* amino acid sequences (Table S1b).

10.1128/mBio.02243-19.5 TABLE S4 Tools used to characterize *Riftia* host and symbiont proteins included in the combined *Riftia* host and symbiont database used in this study. Download Table S4, PDF file, 0.4 MB. Copyright © 2019 Hinzke et al. 2019 Hinzke et al. This content is distributed under the terms of the Creative Commons Attribution 4.0 International license.

### (iii) SMART analysis of eukaryote-like and potential interaction domains.

We used the SMART tool (134) to screen the *Riftia* symbiont protein database for proteins and domains that could be involved in symbiont-host interactions. Structures that did not meet the threshold required by SMART were excluded, whereas overlapping features were included. We manually filtered the SMART annotations to find putative interaction-relevant structures based on Pfam and SMART database information. To compare the *Riftia* symbiont with other host-associated (mutualistic or pathogenic) and free-living organisms, we also included domains not present in the *Riftia* annotations but possibly relevant for host-bacterium interactions in other organisms based on the literature. All annotations we included are given in Table S2. The organisms we used for comparison and their associated proteome accession numbers can be found in Table S1d. Proteins with structures that did not pass the threshold criterion in SMART were removed.

### (iv) Multiple-sequence alignments.

We used the alignment tool MUSCLE, provided by EMBL (https://www.ebi.ac.uk/Tools/msa/muscle/), for multiple-sequence alignment of protein sequences. Alignments were verified visually.

### TEM.

The trophosome sample for TEM was fixed at room temperature for 1 h in fixative containing 4% paraformaldehyde, 1% glutaraldehyde, 10% sucrose in 50 mM HEPES (glutaraldehyde was added directly before use) and stored at 4°C. The sample was washed three times with washing buffer (100 mM cacodylate buffer [pH 7.0], 1 mM CaCl_2_, 0.09 M sucrose) for 10 min each step and treated with 1% osmium tetroxide in washing buffer for 1 h at room temperature. After three additional washing steps in washing buffer for 10 min each, the sample was dehydrated in a graded series of ethanol (30%, 50%, 70%, 90%, and 100%) on ice for 30 min each step. Afterwards, the material was subjected to stepwise infiltration with the acrylic resin LR White according to Hammerschmidt et al. (135). Sections were cut with a diamond knife on an ultramicrotome (Reichert Ultracut, Leica UK Ltd.), stained with 4% aqueous uranyl acetate for 5 min, and finally examined with a transmission electron microscope (LEO 906; Carl Zeiss Microscopy GmbH) at an acceleration voltage of 80 kV. The micrographs were edited using Adobe Photoshop CS6.

### Data availability.

The mass spectrometry proteomics data and the combined host and symbiont database have been deposited to the ProteomeXchange Consortium via the PRIDE (136) partner repository with the data set identifier PXD012439. Transcriptomics raw data have been deposited to the NCBI Sequence Read Archive (https://www.ncbi.nlm.nih.gov/sra) with the BioProject accession number PRJNA534438 (https://www.ncbi.nlm.nih.gov/bioproject/PRJNA534438/).
