# Supplementary material for: Host-Microbe Interactions in the Chemosynthetic Riftia pachyptila Symbiosis
Source: mBio. 2019 Dec 17;10(6):e02243-19. doi: 10.1128/mBio.02243-19 (PMC6918071; doi:10.1128/mBio.02243-19)
Supplement: Text S1 [file mBio.02243-19-s0001.pdf]

Supplementary Online Material

Host-Microbe Interactions in the Chemosynthetic *Riftia pachyptila* Symbiosis

Tjorven Hinzke[1,2,3]*, Manuel Kleiner[3,4], Corinna Breusing[5], Horst Felbeck[6], Robert Häsler[7], Stefan M. Sievert[8], Rabea Schlüter[9], Philip Rosenstiel[7], Thorsten B. H. Reusch[10], Thomas Schweder[1,2], Stephanie Markert[1,2]*

[1] Institute of Marine Biotechnology e. V., Greifswald, Germany

[2] Institute of Pharmacy, Department of Pharmaceutical Biotechnology, University of Greifswald, Germany

[3] Energy Bioengineering Group, University of Calgary, Canada

[4] Department of Plant & Microbial Biology, North Carolina State University, Raleigh, USA

[5] Monterey Bay Aquarium Research Institute, Moss Landing, USA

[6] Scripps Institution of Oceanography, University of California San Diego, San Diego, USA

[7] Institute of Clinical Molecular Biology (IKMB), Kiel University, Kiel, Germany

[8] Biology Department, Woods Hole Oceanographic Institution, Woods Hole, Massachusetts, USA

[9] Imaging Center of the Department of Biology, University of Greifswald, Germany

[10] Marine Evolutionary Ecology, GEOMAR Helmholtz Centre for Ocean Research Kiel, Kiel, Germany

*email addresses:

tjorven.hinzke@outlook.com

stephanie.markert@uni-greifswald.de

Contents

This file:	Supplementary Results and Discussion with Supplementary Figures

The following Supplementary Tables are provided as separate sheets in the Supplementary Excel file:

Supp. Table S1a	All *Riftia* host proteins identified in this study
Supp. Table S1b	Potential autophagy-related *Riftia* proteins
Supp. Table S1c	Potential *Riftia* antimicrobial peptides
Supp. Table S1d	Genomes and metagenomes used for SMART analysis of eukaryote-like protein structures
Supp. Table S1e	Sampling dates, cruise number and number of biological replicates of *Riftia* samples used in this study
Supp. Table S1f	Number of proteins with significant abundance differences in pairwise comparisons of *Riftia* tissues
Supp. Table S1g	All *Riftia* symbiont proteins identified in this study
Supp. Table S1h	Hemerythrin and myohemerythrin isoforms in *Riftia* and other invertebrates used for alignment in Supp. Figure S3
Supp. Table S1i	Carbonic anhydrase isoforms in *Riftia* as detected in this study and described in the literature

The following Supplementary Tables are provided as separate PDF files:

Supp. Table S2	Domains and protein families with a putative role in host-symbiont interactions
Supp. Table S3	Transcriptome completeness for the four different *Riftia* transcriptome assemblies based on the BUSCO eukaryote and metazoan datasets
Supp. Table S4	Tools used to characterize *Riftia* host and symbiont proteins included in the combined *Riftia* host and symbiont database used in this study

Supplementary Results and Discussion

1) General overview

We identified a total of 3,838 *Riftia* host proteins (Supp. Table S1a). Many of these proteins showed statistically significant differences in their relative abundance between tissues (see Supp. Table S1f for total numbers of proteins with significantly different abundances). The "core" proteome of about 700 proteins, which were identified in all host organs under both environmental conditions (i.e. S-rich and S-depleted specimens; Supp. Figure S1), included ribosomal proteins, proteins involved in RNA-, amino acid- and protein metabolism, energy generation, globins, signaling pathways, oxidative stress response as well as cytoskeletal proteins, which are all important throughout the animal. Host proteins only present in the symbiont-bearing trophosome included digestive proteins, stress proteins, sulfate transporters, some histones and proteins involved in amino acid degradation (Supp. Figure S1). Proteins exclusively identified in the plumes included proteins involved in RNA metabolism, apoptosis, carbonic anhydrases, cell division, cytoskeleton and the extracellular matrix, the immune system, signal transduction, and mitochondrial ribosomes. Only identified in the vestimentum were cytoskeletal proteins, proteins of the extracellular matrix, galaxins, proteins involved in chitin degradation and intracellular trafficking (Supp. Table S1a).

Moreover, we identified a total of 1,016 symbiont proteins in the trophosome samples (Supp. Table S1g). Proteins identified in both S-rich and S-depleted symbionts belonged to all main metabolic pathways. Proteins exclusively identified in S-rich symbionts included ribosomal proteins as well as proteins involved in amino acid-, lipid-, and cofactor biosynthesis, nitrogen metabolism, transcription and translation. Proteins only identified in S-depleted symbionts were mostly low-abundant and included mainly hypothetical proteins and a porin. While we did identify a total of 12 symbiont proteins in other tissues than the trophosome, only four of them were present in more than one biological replicate of vestimentum and/or plume samples. These four belonged to the most abundant symbiont proteins (i.e. adenylylsulfate reductase subunits A and B, RubisCO and porin), and are therefore likely contaminations from the dissection procedure. More analyses are necessary to clarify whether the low-abundance proteins are contaminations or proteins secreted by the symbiont.

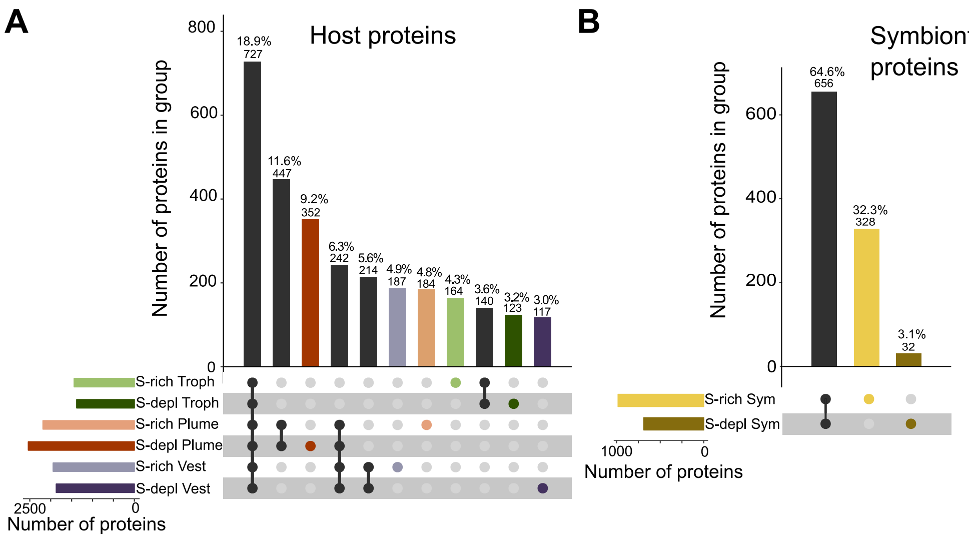

Supp. Figure S1: Overview of the *Riftia* symbiosis metaproteome. A) Intersection plot of the detected *Riftia* host tissue proteins. Bars in the lower left part show the total number of proteins identified in the respective tissue, i.e. in trophosome (Troph), plume or vestimentum (Vest), and condition, i.e. S-depleted (S-depl) or S-rich. Dark/colored dots in the lower right part indicate the samples (single dots) or sample groups (chained dots) in which the proteins are present, equivalent to intersections in a Venn diagram. Columns in the upper part indicate the number and percentage of proteins which are exclusively present in the respective sample or sample group. Shown are selected intersections. B) Intersection plot of *Riftia* symbiont proteins detected in trophosome tissue. Colors in the figures are specific to the respective tissue/symbionts (e.g. yellow for S-rich symbionts, dark green for host proteins from S-depleted trophosome, etc.).

2) Stress reactions and apoptosis in *Riftia* tissues

We identified heat shock proteins and other chaperones as well as universal stress proteins (USPs) of the host in comparatively high total abundances in the trophosome. Four USPs were significantly more abundant in trophosome samples than in the other tissues. Heat shock proteins and other chaperones could be involved in protecting host proteins and DNA in the trophosome against reactive oxygen species (ROS) and toxic sulfur compounds. These toxic compounds could be generated in the course of respiration, symbiont sulfur oxidation and/or symbiont digestion by the host. This would be in accordance with observations in *Bathymodiolus azoricus*, where higher HSP70 levels in the gills than in the mantle were suggested to be a reaction to oxygen and sulfur radicals produced by the symbionts (1). Higher HSP70 levels were furthermore shown to concur with a higher stress tolerance in *Mytilus edulis* (2). While the exact function of USPs is still unknown, they have been observed to be expressed in *Escherichia coli* under various stress conditions (3). In metazoans, proteins with USP domains could have diverse functions in different taxa. USPs of *Hydra* might be involved in protection against microbial infection (4). The *Riftia* USPs could therefore be involved in stress reactions, e.g.

caused by symbiont digestion and subsequent host bacteriocyte death, and in interaction with the symbiont population.

Apoptosis-related proteins, specifically calpains and caspases, on the other hand, were less abundant in the trophosome and rather abundant in the plume: We observed about 4 to 7 times lower levels of calpains in the trophosome than in the plume and vestimentum, and caspases were hardly detected at all in the trophosome, but almost exclusively present in the plume. Apoptosis is probably of higher importance in the plume, as this organ is in direct contact with sulfide-rich water, whereas in the trophosome, another mechanism could be involved in cell death (Main Text). In sulfide-rich environments, ROS are generated (5), which – like sulfide – could cause cell damage, necessitating fast cell turnover. In the trophosome, on the other hand, cell turnover might involve a cathepsin-dependent mechanism (Main Text).

3) Release of organic compounds as a possible mode of nutrient transfer from symbiont to host

Besides symbiont digestion (Main Text), release of organic carbon by intact symbionts ("milking") might also be a relevant mode of nutrient transfer from symbiont to host (6, 7). Our calculation in host tissues and symbionts with Calis-p (8) showed $\delta^{13}C$ values of -6.7 to -8.3 in host tissues, whereas the symbionts where slightly lighter with a $\delta^{13}C$ of -9.8 in S-rich and -11.7 in S-depleted symbionts (Supp. Figure S2). CO_2 fixation via the Calvin cycle yields organic matter with lighter $\delta^{13}C$ values than organic carbon generated by the reverse TCA (rTCA) cycle (9, 10). The $\delta^{13}C$ values we calculated for *Riftia*, which correspond well to those reported in the literature (11), might therefore indicate selective differential export or leakage of small organic compounds derived from the symbiont's rTCA cycle, rather than from the Calvin cycle. On the other hand, the accuracy of the SIF method is limited compared to the isotope ratio mass spectrometry (IRMS) gold standard (8). Additionally, we did not find a dedicated sugar transporter or organic acid exporter in the symbiont proteome and the dissimilar isotopic ratios might also be explained by less discriminating anaplerotic reactions in host tissues, e.g. catalyzed by phophoenolpyruvate carboxykinase (PEPCK), which we detected in all tissues.

Additional circumstantial evidence for milking in the *Riftia* symbiosis might be provided by our comparison of S-rich and S-depleted trophosomes, in which we observed quite dissimilar abundances of digestion enzymes. In S-depleted *Riftia* specimens, symbionts likely secrete less (or

no) carbon compounds than in S-rich specimens, due to reduced CO_2 fixation. Therefore, the host probably needs to increase digestion pressure on the symbionts to cover its own basic nutritional demands. In S-rich specimens, on the other hand, lower digestion rates might be required, because the symbionts leak more organic material to the host. Moreover, it might be possible that the host preferentially digests symbionts with lower S content to avoid high sulfur levels. A similar strategy of preferred digestion of S-depleted symbionts was suggested for the *Codakia orbicularis* symbiosis (12). Whether milking really is a relevant process in the *Riftia* symbiosis remains to be elucidated. Possibly, the *Riftia* host relies on a combination of symbiont digestion and milking, where the relative importance of both processes depends on the environmental conditions. Relative contributions of symbiont digestion and actively released symbiont-derived nutrients to the host's diet may vary considerably in different symbioses, as shown in the *Zoothamnium niveum-Ca.* Thiobios zoothamnicoli symbiosis (13), and in the flatworm *Paracatenula* symbiosis (14).

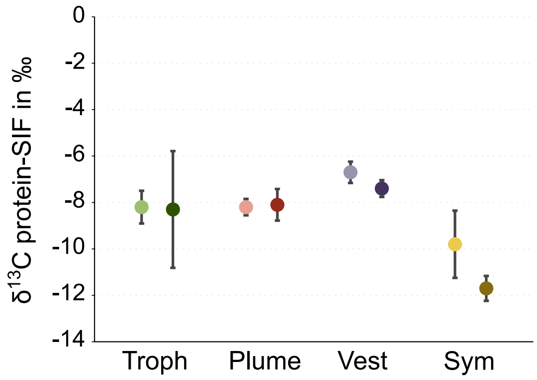

Supp. Figure S2: $\delta^{13}C$ values of *Riftia* host tissues and symbiont cells, calculated from the metaproteomics data using Calis-p (8). SIF: stable isotope fingerprinting. Light-colored symbols indicate S-rich tissues/symbionts, dark symbols indicate S-depleted tissues/symbionts. Error bars indicate standard error of the mean. Troph: trophosome, Vest: vestimentum, Sym: enriched symbiont fraction.

4) Sulfur metabolism in host tissues

Riftia is well known to circumvent toxic effects of sulfide on its eukaryotic tissues by efficiently and reversibly binding sulfide to hemoglobins (15). In addition, the animal may be able to oxidize sulfide in its mitochondria, which would effectively also result in sulfide detoxification. We detected the mitochondrial host enzymes sulfide:quinone oxidoreductase, persulfide dioxygenase, thiosulfate sulfurtransferase and putative thiosulfate sulfurtransferases, which could serve to oxidize sulfide to the less toxic thiosulfate (16). All these enzymes were low-abundant, however. In contrast to other animals in sulfidic habitats, for which mitochondrial sulfide oxidation is the only available

mechanism to avoid sulfide toxicity (17–19), this strategy thus seems to be of minor importance in *Riftia*. For some thiotrophic symbionts, e.g. of *Bathymodiolus* mussels, thiosulfate appears to be the preferred sulfur form (20), and mitochondrial sulfide oxidation to thiosulfate thus likely also promotes symbiont energy generation. As *Riftia* symbionts prefer sulfide (even though they can probably also use thiosulfate; (21–23)), they would presumably not benefit from mitochondrial sulfide oxidation by the host.

Sulfate, produced by the symbionts as end product of sulfide oxidation, could be excreted via the host's sodium-independent sulfate anion transporters detected in the trophosome (after being released from the symbionts with their own sulfate transporter). The host could even use this sulfate for its own biosynthetic processes via a bifunctional 3'-phosphoadenosine 5'-phosphosulfate synthase, which we detected in all vestimentum samples.

5) *Riftia* globins are even more diverse than previously thought

Riftia's extracellular hemoglobins have been thoroughly studied and are well known for their capacity to simultaneously bind O_2 and sulfide ((24–27), reviewed in (15, 28)). In contrast, intracellular *Riftia* globins have received almost no attention thus far. Sanchez et al. (29) reported an intracellular *Riftia* globin, to which none of the globins detected in our study produced a close alignment. This indicates considerable diversity of this hitherto neglected protein group in *Riftia*. Intracellular globins have been described in few other annelid species; only three families are known to have circulating and non-circulating globins in addition to extracellular globins (reviewed in (30)). *Riftia* might possess, in addition to its extracellular hemoglobins, also the other two globin types, which would make it the first described member of the *Siboglinidae* to have this trait: The related siboglinid worm *Lamellibrachia* has coelomocytes (31), indicating that these cells could also be present in *Riftia*. Coelomocytes could contain intracellular, circulating globins, while *Riftia's* intracellular neuroglobins might be non-circulating. Intra- and extracellular globins have also been described in the deep-sea alvinellid *Alvinella pompejana*, where they might buffer against variations in environmental O_2 concentration (32). In vertebrates, intracellular globins like cytoglobin and neuroglobin might be involved in intracellular O_2 storage and transport or may have a protective function against ROS or nitrous oxide, whereas cytoglobin might also have a role in collagen production (33). The role of *Riftia's* intracellular globins awaits further testing.

6) *Riftia* myohemerythrins as possible multi-purpose proteins

We detected myohemerythrins as additional possible O_2-binding molecules besides the well-described hemoglobins in *Riftia*. Hemerythrins are oxygen-binding molecules that can bind 15-28% more oxygen than simple hemes (34). Myohemerythrin is hemerythrin localized in (annelid) muscle cells (reviewed in (35)).

Our protein analysis showed a tissue-specific abundance pattern of the nine detected *Riftia* myohemerythrins. We therefore analyzed the sequences of these proteins to find indications for their respective functions. Additionally, we included hemerythrin sequences of annelids, a brachiopod and a member of the Bryozoa from the NCBI database (https://www.ncbi.nlm.nih.gov/) in our comparison (Supp. Figure S3, Supp. Table S1h). Almost all of the conserved sites that exist in hemerythrins (as outlined by Stenkamp (36)) are present in most *Riftia* myohemerythrins. One residue likely involved in O_2 binding has been described to either be a leucine or an isoleucine (36). This residue corresponds to residue 76 (the positions refer to *Riftia*_myoHr6 in Supp. Figure S3), which is an isoleucine in most of the *Riftia* myohemerythrins, but a leucine in two sequences, both of which are significantly less abundant in the plume than in the trophosome samples. In the same sequences, residue 117 has been replaced by phenylalanine. This replacement has been noted before (37) for one *Riftia* myohemerythrin sequence (ABW24415.1), which corresponds to Host_DN34237_c2_g1_i1::g.35857 analyzed in our study (Host_DN34237_c2_g1_i1::g.35857 is just 10 amino acids longer at the N terminus). Additionally, the six myohemerythrins with significantly higher abundance in the plume have several identical regions which differ from at least two of the other three myohemerythrins (Supp. Figure S3). While those are not assigned a specific function and we can therefore only speculate about their biological effect, these different variants nevertheless point to functional, tissue-specific diversification of the *Riftia* myohemerythrins. Possibly, the myohemerythrins in the trophosome are involved in interactions with the symbionts, whereas the plume-located myohemerythrins play a more general role in O_2 metabolism. A trophosome-specific *Riftia* myohemerythrin analyzed by Sanchez (37) was suggested to be involved in protection against O_2 radicals, especially as potentially high radical production occurs in the trophosome. While reversible O_2 binding by an overexpressed *Riftia* myohemerythrin could not be shown by Sanchez (37), this does not rule out *Riftia* myohemerythrin functioning as an O_2 carrier, as it is apparently not uncommon that purified hemerythrins do not bind O_2 (38). Myohemerythrins have been noted to fulfill similar functions as myoglobin, which generate an O_2 gradient to the mitochondria (39). Heart and skeletal muscle cells as well as rhizobium nodules use myoglobin or leghemoglobin, respectively,

to transport O_2 to the mitochondria/bacteroids. Leghemoglobin additionally likely protects the bacteroids against O_2 diffusion inside the cells, which contain the highly O_2-sensitive nitrogenase (reviewed in (40)). *Riftia* myohemerythrin in the trophosome could fulfill a similar function in provisioning the symbionts with O_2 while protecting them from free O_2 (41). This might also enable the symbionts to use the O_2-sensitive rTCA cycle while respiring O_2. Additionally, the *Riftia* myohemerythrin in the trophosome could be involved in regulating symbiont access to metals. For a hemerythrin-like protein in *Nereis diversicolor*, for example, it has been suggested that it regulates cadmium levels, as it binds cadmium in the gut (42).

Riftia myohemerythrins which are more abundant in the plume might be involved in maintaining an O_2 gradient across the plume and thereby facilitate oxygen diffusion into the animal. Additionally, they could store oxygen, as suggested for sipunculans during low tides (34) and as proposed for the symbiotic oligochaete *Olavius algarvensis* (43).

Moreover, *Riftia* myohemerythrins in general might serve further purposes as observed in other animals, e.g. iron storage, metal detoxification and functions in the immune system (37–39, 44). Potentially, they might even be involved in nitrate transport, as nitrate binding and concentrating by *Riftia* blood was observed (45). The exact function and (sub-) cellular localization of the different myohemerythrins deserves future studies, especially as an association with muscle cells in the non-muscular trophosome tissue seems unlikely. We rather speculate that myohemerythrin is contained in host bacteriocytes.

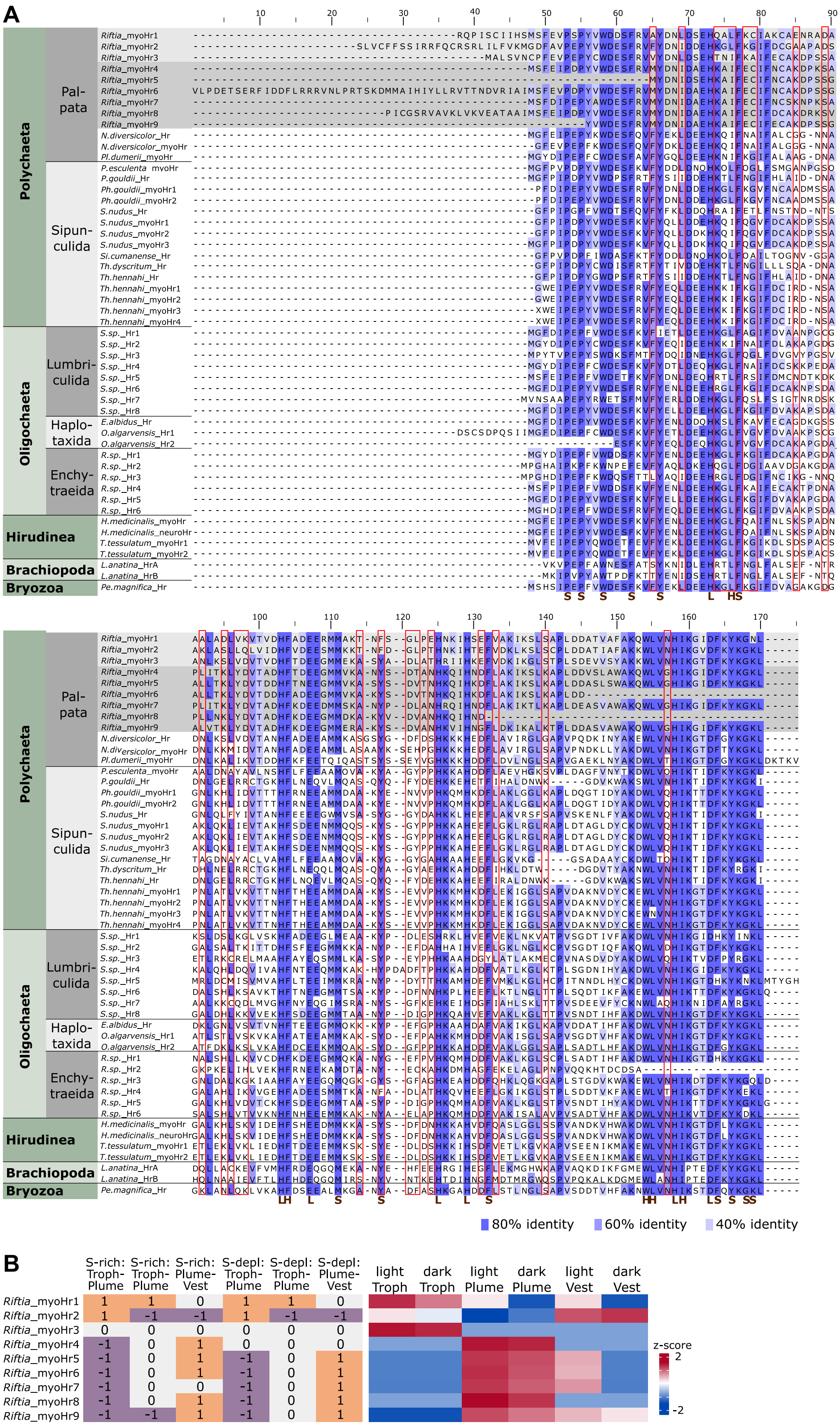
A
Polychaeta
Palpata
Sipunculida
Oligochaeta
Lumbriculida
Haplotaxida
Enchytraeida
Hirudinea
Brachiopoda
Bryozoa
80% identity
60% identity
40% identity
B
S-rich: Troph-Plume
S-rich: Troph-Plume
S-rich: Plume-Vest
S-depl: Troph-Plume
S-depl: Troph-Plume
S-depl: Plume-Vest
light Troph
dark Troph
light Plume
dark Plume
light Vest
dark Vest
Riftia_myoHr1 1 1 0 1 1 0
Riftia_myoHr2 1 -1 -1 1 -1 -1
Riftia_myoHr3 0 0 0 0 0 0
Riftia_myoHr4 -1 0 1 0 0 0
Riftia_myoHr5 -1 0 1 -1 0 1
Riftia_myoHr6 -1 0 1 -1 0 1
Riftia_myoHr7 -1 0 0 -1 0 1
Riftia_myoHr8 -1 0 1 -1 0 1
Riftia_myoHr9 -1 -1 1 -1 0 1
z-score
2
-2

Supp. Figure S3: A) Alignment of *Riftia* myohemerythrins with myohemerythrins of various other invertebrates (Supp. Table S1h). Conserved residues according to Stenkamp (36) are marked as follows: L: iron ligand binding, S: suggested to have a structural role, H: hydrophobic residues in O_2-binding pocket. Red boxes denote residues which are identical in one *Riftia* myohemerythrin subgroup (subgroups were separated by their distinct abundance patterns and are shown with different grey backgrounds) and different from at least two thirds of the sequences in the other subgroup. B) Left side, significance of abundance differences of detected myohemerythrins in *Riftia* tissues. 1: Significantly more abundant in the first tissue than in the second tissue, -1: significantly less abundant in the first than second tissue, 0: no significant difference. Right side, heatmap of the z-scored %orgNSAF abundance of myohemerythrins in the different *Riftia* tissues. For accession numbers, see Supp. Table S1h.

7) *Riftia* carbonic anhydrases are tissue-specific

Riftia expresses abundant carbonic anhydrases (CA) for interconversion of CO_2 and HCO_3^- (Main text). Apparently, distinct isoforms dominate in different tissues (Main Text, Supp. Figure S4). Tissue-specific expression of *Riftia* CA has been noted before (46, 47). Three of the CAs detected in our study align closely to the CAs analyzed in these previous studies (Supp. Figure S4). Previous biochemical experiments hinted towards the existence of membrane-bound *Riftia* CA (47, 48). Indeed, predicted transmembrane helices and/or signal peptides in three of the CA sequences in our study indicate that these could be membrane-bound (or even secreted) rather than cytoplasmic. These three CAs also align more closely to each other than to the other CA sequences (Supp. Figure S4). Interestingly, putative membrane-bound CA isoforms seem not to be restricted to one tissue, but might increase CO_2 transport efficiency across different tissues. The residues involved in zinc binding and several of the residues forming the hydrogen bond of the active site according to De Cian et al. (47) seem not to be conserved across all *Riftia* CA isoforms, indicating possible functional diversification.

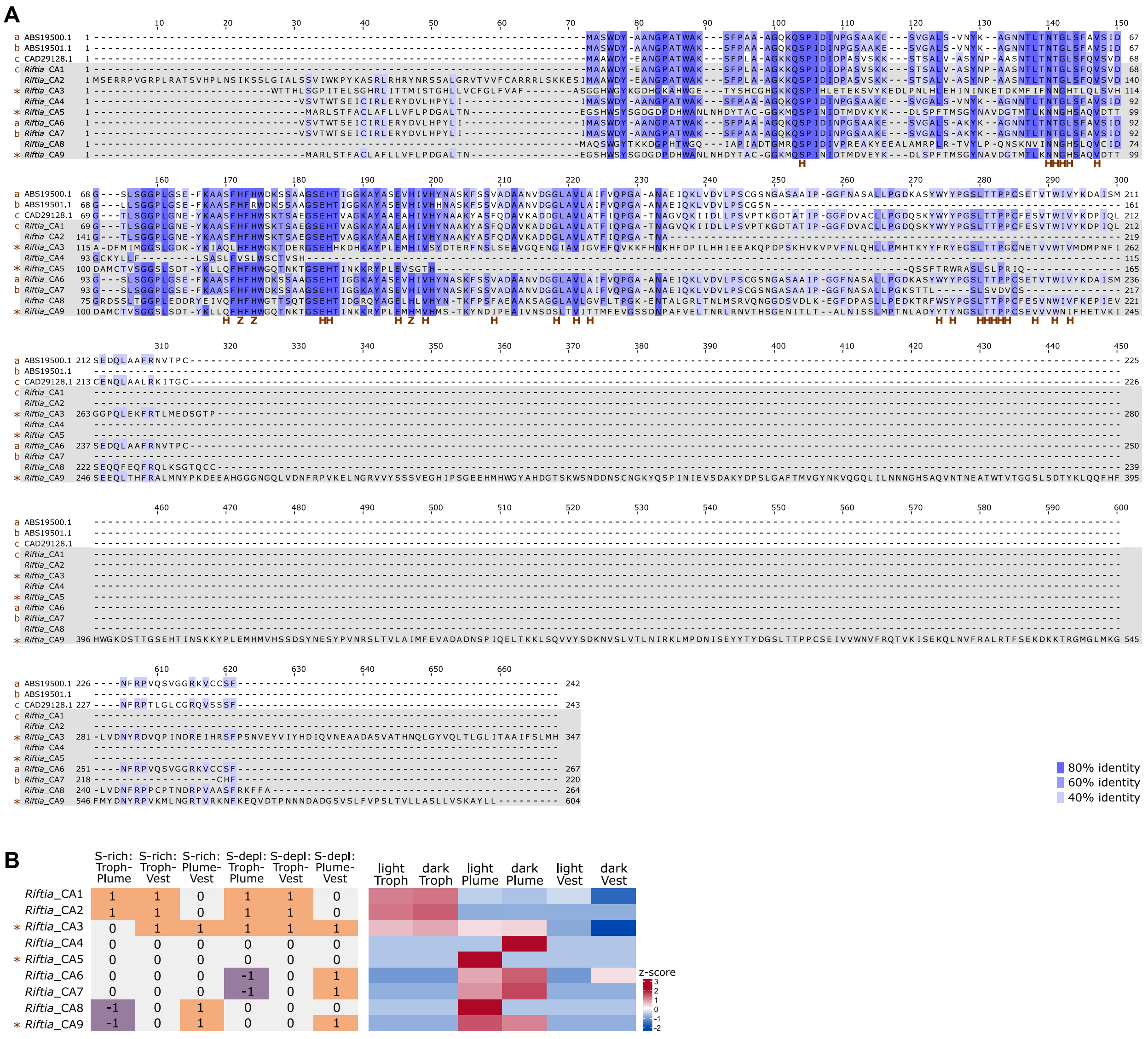

Supp. Figure S4: A) Alignment of carbonic anhydrases (CAs) detected in this study (Riftia_CA1 – Riftia_CA9) and CAs detected in previous studies ((46, 47), for accession numbers see Supp. Table S1i). Lower case letters indicate which CA sequences identified in our study align most closely to the sequences identified in previous studies. Asterisks mark potentially membrane-bound/secreted CA isoforms. Capital letters mark residues involved in functions according to De Cian et al. (47). H: residues forming the hydrogen bond network of the active site, Z: histidine residues involved in zinc binding. B) Left side, significance of abundance differences of detected CAs in the *Riftia* tissue comparisons. 1: Significantly more abundant in the first tissue than in the second tissue, -1: significantly less abundant in the first than second tissue, 0: no significant difference. Right side, heatmap of the z-scored %orgNSAF abundance of CA in the different *Riftia* tissues. For accession numbers, see Supp. Table S1i.

8) C_4 metabolism in the *Riftia* host?

We detected proteins involved in transport of inorganic carbon by the *Riftia* host to its symbiont, i.e., V-type ATPase subunits, carbonic anhydrase and bicarbonate exchangers (Main Text Figure 2). In

addition, it has previously been suggested that the host also provides the symbionts with pre-fixed CO_2, i.e., with small organic compounds (49, 50). Phosphoenolpyruvate carboxykinase, which we detected in all host tissues, and pyruvate carboxylase, which was only lacking in S-rich plumes (Supp. Table S1a), catalyze incorporation of CO_2 into oxaloacetate, which would then be available for host and symbiont metabolism. Like for the *Riftia* symbiosis, host pre-concentration of CO_2 via phosphoenolpyruvate carboxykinase has been postulated for the *B. azoricus* symbiosis (20). On the other hand, pre-fixation of CO_2 in the *Riftia* symbiosis has been doubted by Childress et al. (51), who observed high levels of total CO_2 and CO_2 partial pressure in the blood of the tubeworm. Following this, ^{14}C-labelled malate and succinate formation in the plume observed by Felbeck (49) could also be due to anaplerotic host metabolism, rather than presenting a means to sustain the symbionts. If host pre-fixation of CO_2 would occur, the symbiont would either need transporters for uptake of the organic compounds, or the organic compounds would needed to be decarboxylated inside the host cells, so that the symbiont could take up CO_2. We found five symbiont TRAP transporter components in the trophosome samples, two of which were predicted to transport C_4 dicarboxylates, which could take up small organic CO_2 carriers. At the same time, import of these organic CO_2 transport molecules into the bacteriocyte (and maybe the symbiont cell) would probably rule out simultaneous leakage or export of small organic molecules by the symbiont to sustain the host (milking, see above), as uptake and concomitant release of small organic molecules by the bacteriocyte seems unlikely. Therefore, it remains speculative whether the *Riftia* host pre-fixes CO_2, also in light of our metaproteomics data.

9) Nitrogen metabolism in the *Riftia* symbiosis

Our reconstruction of nitrogen metabolic pathways in the *Riftia* host and symbiont revealed that *Riftia* likely depends on ammonium supplied by the symbiont, but can synthesize pyrimidines and polyamines independently (main text, detailed below), while the host might degrade nitrogen-containing symbiont waste products.

We found two possible NO_3^- transporters (probable peptide/nitrate transporter At3g43790: Host_DN35983_c1_g1_i7::g.173229, Host_DN35983_c1_g1_i4::g.173222) in the *Riftia* transcriptomes of all tissues, although not in the metaproteome. The respective proteins could be involved in NO_3^- uptake from the environment and NO_3^- transport inside the animal. Hypothetically, *Riftia* myohemerythrin, which was abundantly detected in the host metaproteome, might serve as a nitrate-

binding component in the blood, in addition to hemoglobin, which has previously been suggested to bind and concentrate NO_3^- (45). After transport to the symbionts, nitrate could be ammonified via the symbiont's membrane-bound nitrate reductase (NarGHIJ) and nitrite reductase (NirBD) (Main Text Figure 4). Although NarGHIJ is mainly known to be involved in dissimilatory nitrate reduction, i.e., respiration, assimilatory nitrate reduction via membrane-bound NarGHJI and NirBD was demonstrated in *Mycobacterium tuberculosis* (52) and suggested for the symbiont of the tubeworm *Ridgeia piscesae* (53). Symbiont periplasmic nitrate reductase NapAB, of which we detected NapB in the metaproteome, could theoretically also be involved in assimilatory nitrate reduction ((53), reviewed in (54)). Both partners of the *Riftia* symbiosis could then incorporate ammonium into their organic matter via glutamine synthetase/glutamate synthase (GS-GOGAT cycle) as well as via glutamate dehydrogenase, all of which we detected on the protein level in host and symbiont. Host glutamate dehydrogenase was overall most abundant in the trophosome, compared to other tissues, possibly because the host metabolizes ammonia generated by the symbionts. Eukaryotic ammonium assimilation via the GS-GOGAT cycle and via glutamate dehydrogenase has previously been suggested in silkworms (55, 56), mosquitoes (57) and *R. piscesae* tubeworms (53).

Both symbiotic partners appear to be capable of producing the polyamine putrescine (Main Text Figure 4). This contradicts a previous, enzyme activity-based study, in which arginine decarboxylase and ornithine decarboxylase activity were presumed to be exclusively due to symbiont enzymes (58). Given the ubiquitous importance of polyamines (see below), it is probably advantageous for the host to be less dependent on the symbiont for polyamine synthesis, even more so during times of reduced symbiont digestion (i.e. in S-rich specimens, Main Text).

Our results suggest that *Riftia* degrades nitrogenous compounds via the urea cycle. We detected all urea cycle enzymes (except for nitroxide synthase and arginine deaminase, which are not required for the full cycle) in the host's proteome, although not all proteins were detected in all tissues. We also identified a urea-proton symporter, probably involved in urea excretion, in all plume samples and one vestimentum sample. Interestingly, the symbiont, unlike the host, does not seem to possess a complete urea cycle (Main Text Figure 4). As host arginase was significantly more abundant in the trophosome than in the other tissues, it probably serves to break down nitrogen-containing metabolites of host and symbiont. Taken together, our results point to a complete urea cycle in the *Riftia* trophosome, as previously indicated by biochemical analyses (59).

10) Host polyamines may be involved in host-symbiont interactions

We detected host spermine synthase and spermidine synthase, which synthesize the polyamines spermine and spermidine, only in the trophosome metaproteomes, but not in other tissues. Therefore, *Riftia* polyamines could have a role in symbiont-host interactions. Such a role for a polyamine has been suggested for *Shigella flexneri*, where cadaverine is likely involved in restricting the pathogen to the host phagocytic vacuole (60). Similarly, *Riftia* host polyamines could be involved in keeping the symbiont from spreading in the host tissue. Additionally, spermine could play a role in immune system suppression in the trophosome, as it has been shown to suppress an innate inflammation reaction in human cell cultures after lipopolysaccharide stimulation (61). In addition to these putative interaction-related functions, the polyamines of both *Riftia* host and symbiont probably also have more general roles: Polyamines are known to interact with DNA, RNA and proteins, are involved in reaction to oxidative and acid stress and apoptosis, and play a role in bacterial outer membrane biogenesis and functions. Polyamine levels are higher in actively proliferating cells (reviewed in (62–64)). This may explain the high abundance of polyamine-generating enzymes in *Riftia* trophosome, where host and symbiont cells proliferate at very high rates in the central parts of the trophosome lobules (65).

11) High FIH abundance points to hypoxic conditions in the *Riftia* trophosome

Our results indicate that *Riftia* sustains hypoxic conditions in the trophosome, which would benefit the microaerophilic symbionts. The hypoxia-inducible factor 1-alpha inhibitors (factor inhibiting HIF1a; FIH), which we detected almost exclusively in trophosome samples, are possibly involved in metabolic regulation under these conditions. FIH inhibits HIF1a, which is a main regulator of hypoxia-dependent transcription regulation, during normoxic conditions (66). While we did not detect HIF1a on the protein level, the presence of FIH very likely indicates that HIF1a is also expressed. A low (but detectable) abundance of FIH might indicate that HIF1a is not inhibited and the trophosome is hypoxic. High FIH abundance, on the other hand, would indicate HIF1a inhibition and thus more normoxic conditions, e.g. during temporary phases of higher O_2 concentration. The hypothesis of a hypoxic trophosome is in line with the observation that *Caenorhabditis elegans* HIF1a is induced by hypoxia and rapidly degraded under normoxic conditions (67). Further studies are needed to verify the presence of HIF1a and to elucidate the regulation of FIH itself under different O_2 regimes in *Riftia* trophosomes.

12) The *Riftia* immune system might protect the symbiosis against phages

The *Riftia* symbionts appear not to elicit a general host immune response, as a number of host immune proteins such as lysozyme, tyrosine-protein kinases and peptidoglycan recognition proteins were detected, but overall, their abundance in the trophosome was not higher than in other tissues (Main Text Figure 2). Galaxins, proteins associated with coral exoskeletons (68), have been implied in immune system and symbiont interaction functions in squids (69). While one galaxin transcript was detected before in the *Riftia* body wall (29), we only detected galaxins in the firm vestimentum tissue, implying that they could have a structural rather than an immune system function in *Riftia*. Most likely, the host vesicles in which the symbionts are contained shield the bacteria against recognition and attack by the *Riftia* immune system. Similarly, in chronic infections of insect hosts with endosymbionts, the host's immune system does not eliminate the symbionts (70). Rather than acting against the symbionts, the *Riftia* immune system probably protects the host (and thereby the symbiosis as a whole) against infections, and might even be involved in protecting the symbionts against phage infections. The symbiont population is basically a bacterial monoculture inside *Riftia*, for which phage infections could easily be fatal. The detection of CRISPR-Cas-associated proteins in the *Riftia* symbiont metaproteome might indicate that phage infections play a role during symbiosis. Symbionts could be prone to infection also inside the trophosome, as phages can rapidly penetrate tissues of higher organisms (71). Therefore, host proteins likely involved in interaction with viruses, e.g. interferon-induced GTP-binding proteins, glycylpeptide N-tetradecanoyltransferase 2 and deoxynucleoside triphosphate triphosphohydrolase SAMHD1, could not only protect *Riftia* itself against a viral infection, but could putatively also be involved in shielding the symbionts against phages. The *Riftia* immune system could also be involved in initial identification of the symbiont during host colonization (e.g. via lectins which are present in the host's proteome). In the symbiotic stage, however, few and specific functions of the immune system seem to be harnessed for symbiotic interactions.

13) Roles of eukaryote-like proteins in *Ca.* E. persephone and other host-associated and free-living organisms

We compared the proportion of proteins with putative roles in host-symbiont interactions, especially eukaryote-like proteins (ELPs), which are encoded in the metagenomes of several (endo)symbionts, pathogens, free-living bacteria and environmental samples (Main Text Figure 5, Supp Figure S5,

Supp. Table S2, Supp. Table S1d). The three *Riftia*/*Tevnia* symbiont metagenomes that were included in our protein database (see methods) showed, as expected, almost identical distribution patterns of the respective protein groups. As *Riftia* and *Tevnia jerichonana* are colonized by the same symbiont species (72, 73), differences are probably due to unequal sequencing coverage. The pattern observed in the *Ridgeia* symbiont metagenomes was very similar to that in the *Riftia/Tevnia* symbiont, which is in line with the fact that the *Ridgeia* symbiont likely belongs to the same species as the *Riftia* and *Tevnia* symbiont (73). The similarity of ELP patterns between the tubeworm symbionts and symbionts of shallow-water bivalves (Supp. Figure S5) suggests parallels in interaction strategies with their respective hosts.

We also observed differences in ELP distribution patterns between distinct organism groups (Supp. Figure S5), which points to specific environmental adaptations. Armadillo repeats, FG-GAP, GIDE, Hyr, Integrins, and TSP domains were almost exclusively found in the gammaproteobacterial sulfur-oxidizing endosymbionts, suggesting specialized roles in these associations. The low number of the respective genes in the *B. azoricus* sulfur-oxidizing endosymbiont metagenomes could be an artifact caused by the small and presumably rather incomplete metagenome databases available. On the other hand, whereas *Riftia* endosymbionts are probably actively digested by the host – and even more so in times of starvation – in *B. azoricus*, autolysis of senescent bacteria seems to be part of the normal cell cycle (74). This could explain a higher abundance of putative host interaction-related and presumably phagocytosis-inhibiting proteins in the *Riftia* symbiont, and in the symbionts of the shallow-water bivalves, which are probably also actively digested by their hosts (75–77). The *B. thermophilus* endosymbiont ELP distribution pattern was rather distinct from that of all other gammaproteobacterial SOX endosymbionts. The reasons for these differences, including especially the high abundance of attachment-related proteins in the *B. thermophilus* endosymbiont, remain open for debate.

Some domains, especially ankyrins, PD40 and WD40, Sel1, SNARE, TPR and coiled-coil domains are rather widespread and are also found in non-host-associated bacteria. This points to more general functions, which might have been harnessed in the course of symbiotic adaptations. Some of the analyzed host-associated bacteria, including the *Riftia* symbiont, are horizontally transmitted, i.e. taken up from the environment (78, 79). This could explain the relatively high similarity of these bacteria to the protein group pattern of free-living organisms and the diversity of protein groups. In comparison, obligate endosymbionts (including *Calyptogena* endosymbionts (80)), which undergo genome reduction and genetic drift, showed a reduced diversity of the analyzed protein groups.

Nipsnap domains are mainly found in rhizobia and could thus be involved more specifically in rhizobia-plant interactions. As expected, the vent- and chimney metagenomes, which encompass a variety of prokaryotic and eukaryotic microorganisms, had the highest total number of eukaryote-like domain-containing proteins. Nevertheless, relative to the total number of proteins in the respective (meta)genomes, some of these protein groups are under-represented in comparison to the single bacteria analyzed, which indicates that ELP patterns may vary substantially between microbes and appear to have specific functions in individual bacterial groups.

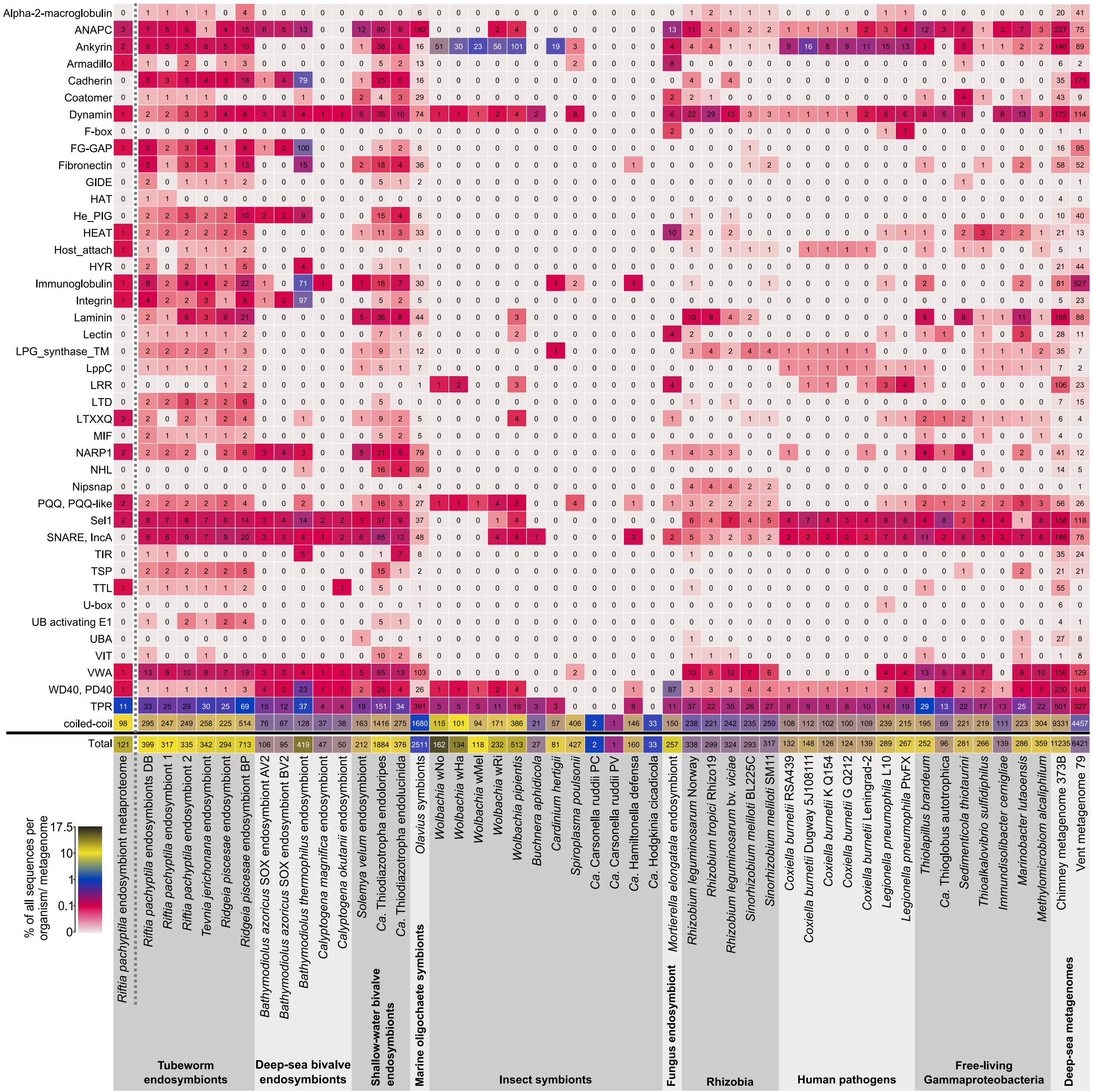

Supp. Figure S5: Selected domains with eukaryote-like structures and with putative functions in symbiont-host interactions in the *Riftia* symbiont and in selected other organism groups and environmental metagenomes. Color scale: percentage of genes/proteins with the respective domain relative to all genes/proteins, numbers: total number of genes/proteins with the respective domain. For details on the organisms, see Supp. Table S1d. For further information about the selected protein groups, see Supp. Table S2. '*Riftia pachyptila* endosymbiont metaproteome' refers to *Riftia* symbiont proteins detected in this study.

14) Symbiosis-relevant proteins of *Ca.* E. persephone may facilitate host interactions

Symbiont proteins might modulate host gene expression. Our metaproteomic analyses suggest that host histones could interfere with the symbiont, because they can be precursors for antimicrobial peptides (AMPs, Main Text). The symbiont, on the other hand, could itself manipulate the host's histones, and thereby the host transcription regulation: We found the SET domain-containing protein Sym_EGV51182.1 in the *Riftia* symbiont's metagenome (albeit not on the protein level). Sym_EGV51182.1 had highest sequence similarity to several eukaryotic histone-lysine N-methyltransferases with 30-32% identity (BLASTP against Uniprot/Swissprot, https://www.expasy.org/). In *L. pneumophila*, a lowly expressed SET domain-containing methyltransferase trimethylates host histone H3 and thereby probably represses the expression of host genes, including genes of the innate immune system (81). The *Riftia* symbiont SET domain-containing protein might have a similar function and modulate gene expression of the host, possibly enabling symbiont persistence by repressing the host's immune response.

***Ca.* E. persephone dodecin and porin may facilitate symbiont survival.** We detected a putative symbiont dodecin (Sym_2601633487) in about 30 times higher abundance in symbionts from S-depleted trophosomes (0.886%orgNSAF, rank 16) than in symbionts from S-rich trophosomes (0.028%orgNSAF, rank 409). This protein could be involved in adaptation to conditions under S depletion, e.g. to a putatively increased digestion pressure. A BLAST search of the *Ca.* E. persephone putative dodecin against the Uniprot/Swissprot database (https://web.expasy.org/blast/) showed highest similarity to a *Halorhodospira halophila* dodecin (54% identity), followed by a *Thermus thermophilus* dodecin (45% identity). *H. halophila* dodecin was shown to bind the flavin FMN (82). Likewise, the dodecin of *T. thermophilus* binds FMN and coenzyme A. Dodecin could therefore work as a flavin trap, for example when flavins are released from denatured flavoproteins (83). Reduced flavins and flavoproteins generate ROS (84). The *Ca.* E. persephone dodecin could therefore counteract the detrimental effects of putatively higher oxygen levels in S-depleted trophosomes by

sequestering flavins, especially as probably less flavins are needed for redox reactions and electron transfers and additionally flavoproteins might be degraded due to the energy-limited conditions. Higher stress levels due to digestion could also facilitate flavin release from symbiont proteins. Sequestering these flavins by dodecin would decrease the potential for ROS formation. Specific involvement of dodecin in intracellular survival has been speculated for *M. tuberculosis*. This protein could be involved in survival of the pathogen under the acidic conditions in lysosomes, as the FMN:dodecin complex is more stable at a lower pH (85). Likewise, the *Riftia* dodecin could exert a protective influence during increased symbiont digestion in S-depleted trophosomes.

Besides dodecin, symbiont porins, the most abundant symbiont proteins, were also notably (about four times) more abundant in S-depleted symbionts (9.8%orgNSAF) than in S-rich symbionts (2.4%orgNSAF). Porins could be involved in interactions with the host by exporting symbiont effectors, or by im- or exporting host effectors, e.g. to modulate digestion (see also Main Text). Their higher abundance in S-depleted trophosomes could therefore be a reaction to the postulated higher digestion pressure. In the genetic neighborhood of the *Riftia* symbiont porin (Riftia1 genome) are several genes with predicted functions in lipopolysaccharide biosynthesis, and sugar metabolism and RNA metabolism. However, the exact function of the *Ca.* E. persephone porin remains to be elucidated.

***Ca*. E. persephone might be naturally competent during symbiosis.** Several of the symbiont proteins detected in this study indicate that the *Riftia* symbiont population is genetically competent during symbiosis and might exchange genetic material. The type 4 pilus (T4P) system might be involved in natural transformation (Main Text). Besides the T4P system, we identified proteins belonging to toxin-antitoxin-systems (putative zeta toxins Sym_EGV52816.1, Sym_EGW56021.1). These can be encoded on conjugative elements, so that bacteria which do not have these conjugative elements are killed (86). Additionally, proteins of the Tol-Pal-system were present in the *Ca*. E. persephone metaproteome. This system not only confers outer membrane stability, but is also involved in uptake of the mostly plasmid-encoded bacteriocin colicin A in *E. coli* (87, 88). Moreover, we detected two plasmid-related proteins (Sym_EGV50018.1 – plasmid maintenance system killer, Sym_EGV50285.1 – TraB family pAD1 protein). Previous genome analyses showed that the *Riftia* symbiont has an integrated partial F plasmid (Riftia1 genome; (72)). Conjugation could allow the symbionts to exchange genetic material, potentially with a role in symbiosis. These results indicate

that strain adaptation and diversification of the *Riftia* symbiont might take place under symbiotic conditions.

15) Metabolite exchange between the symbiosis and the environment depends on S availability

Lower S availability seems to not only impact the *Riftia* host's nutrition, but also its exchange of compounds with the environment and thereby its capacity to support the symbiont: We detected carbonic anhydrases (CAs) and FIH in higher total abundance in S-depleted specimens (summed %orgNSAF), whereas V-type ATPase subunits, myohemerythrin and cytoplasmic malate dehydrogenase were overall less abundant in S-depleted specimens. These differences are probably due to changes in the habitat chemistry: Sites with low sulfide concentrations (leading to low S availability for the symbiosis) are alkaline, whereas those with higher sulfide levels have an acidic to neutral pH (89). At the same time, higher sulfide concentrations implicate higher CO_2 levels, but lower O_2 concentrations. In plumes of *Riftia* specimens from habitats with low sulfide levels (and thus also low CO_2 levels), higher activities of carbonic anhydrase where measured (89). The higher abundances of carbonic anhydrases in trophosomes and plumes of S-depleted specimens thus probably serve to enable CO_2 uptake against a shallower concentration gradient (if CO_2 is taken up at all under these conditions). Moreover, as autotrophic metabolism of the symbionts is probably inhibited during sulfide depletion (90), CO_2 generated by host and symbiont respiration would need to be excreted in order to prevent metabolic acidosis. CA could then function to catalyze the conversion of bicarbonate to CO_2 to facilitate CO_2 diffusion into the environment. At the same time, the need for pH regulation seems to be decreased in S-depleted *Riftia*, as we detected a lower sum abundance of V-type ATPase subunits in plumes of these specimens. In S-depleted worms, less protons are generated during symbiont sulfide oxidation, which diminishes the need for ATP-consuming proton excretion.

Whereas reduced sulfur compounds, and potentially CO_2, are probably limiting under S-depleted conditions, O_2 could be limiting in S-rich specimens: Myohemerythrin, which is potentially involved in O_2 storage and transport, had an about 2.9 times higher overall abundance in S-rich plumes and trophosomes than in S-depleted plumes and trophosomes. It might facilitate O_2 uptake and O_2 provision of the symbionts, which are relatively more abundant, and thus in total likely need more O_2, in S-rich trophosomes. At the same time, trophosomes of S-rich specimens apparently still have

lower free O_2 levels, as indicated by about 10 times lower levels of FIH in S-rich trophosomes and higher malate dehydrogenase abundance (see above and Main Text). Although, on the one hand, hypoxic conditions would be beneficial for the microaerophilic symbionts, on the other hand, too low O_2 concentrations could limit sulfur oxidation and add to elemental sulfur accumulation in symbionts of S-rich *Riftia* specimens. While the symbionts are possibly capable of NO_3^- respiration, this pathway probably plays only a minor role compared to O_2 respiration (91). Additionally, the energy gained from NO_3^- respiration would be lower than that from aerobic respiration, due to the lower redox potential of NO_3^-. The higher myohemerythrin abundance thus likely points to an increased O_2 demand in S-rich *Riftia* specimens. Similarly, myoglobin concentrations in vertebrate muscles increase with oxygen demand, and myoglobin expression depends at least partially on O_2 availability (reviewed in (40)).

The symbiont metaproteome profile also hints at more oxic conditions in S-depleted than S-rich trophosome: Two of the symbiont antioxidant proteins (Sym_EGV49864.1 – rubrerythrin, Sym_EGV52457.1 – putative hemerythrin) were significantly more abundant in the S-depleted symbionts. Moreover, it could be speculated that the symbionts are killed before digestion via an oxidative burst, as is the case for intracellular pathogenic bacteria (92). The higher antioxidant levels in S-depleted symbionts would then be a means to counteract the host-produced ROS. A role of hemerythrin for survival inside host macrophages via ROS detoxification has been suggested for *Aeromonas hydrophila*, a gram-negative gammaproteobacterium (93).

References

1. Pruski AM, Dixon DR. 2007. Heat shock protein expression pattern (HSP70) in the hydrothermal vent mussel *Bathymodiolus azoricus*. Mar Environ Res 64:209–224.
2. Tedengren M, Olsson B, Reimer O, Brown DC, Bradley BP. 2000. Heat pretreatment increases cadmium resistance and HSP 70 levels in Baltic Sea mussels. Aquat Toxicol 48:1–12.
3. Kvint K, Nachin L, Diez A, Nyström T. 2003. The bacterial universal stress protein: function and regulation. Curr Opin Microbiol 6:140–145.
4. Forêt S, Seneca F, de Jong D, Bieller A, Hemmrich G, Augustin R, Hayward DC, Ball EE, Bosch TCG, Agata K, Hassel M, Miller DJ. 2011. Phylogenomics reveals an anomalous distribution of USP genes in metazoans. Mol Biol Evol 28:153–161.
5. Tapley DW, Buettner GR, Shick JM. 1999. Free radicals and chemiluminescence as products of the spontaneous oxidation of sulfide in seawater, and their biological implications. Biol Bull 196:52–56.
6. Bright M, Keckeis H, Fisher CR. 2000. An autoradiographic examination of carbon fixation, transfer and utilization in the *Riftia pachyptila* symbiosis. Mar Biol 136:621–632.
7. Felbeck H, Jarchow J. 1998. Carbon release from purified chemoautotrophic bacterial symbionts of the hydrothermal vent tubeworm *Riftia pachyptila*. Physiol Zool 71:294–302.
8. Kleiner M, Dong X, Hinzke T, Wippler J, Thorson E, Mayer B, Strous M. 2018. Metaproteomics method to determine carbon sources and assimilation pathways of species in microbial communities. Proc Natl Acad Sci U S A 115:E5576–E5584.
9. Pearson A. 2010. Pathways of carbon assimilation and their impact on organic matter values $\delta^{13}C$, p. 143–156. *In* Timmis, KN (ed.), Handbook of Hydrocarbon and Lipid Microbiology. Springer, Berlin, Heidelberg.
10. Hügler M, Sievert SM. 2011. Beyond the Calvin cycle: autotrophic carbon fixation in the ocean. Ann Rev Mar Sci 3:261–289.
11. Fisher CR, Kennicutt II MC, Brooks JM. 1990. Stable carbon isotopic evidence for carbon limitation in hydrothermal vent vestimentiferans. Science 247:1094–1096.
12. Caro A, Gros O, Got P, De Wit R, Troussellier M. 2007. Characterization of the population of the sulfur-oxidizing symbiont of *Codakia orbicularis* (Bivalvia, Lucinidae) by single-cell analyses. Appl Environ Microbiol 73:2101–2109.
13. Volland J-M, Schintlmeister A, Zambalos H, Reipert S, Mozetič P, Espada-Hinojosa S, Turk V, Wagner M, Bright M. 2018. NanoSIMS and tissue autoradiography reveal symbiont carbon fixation and organic carbon transfer to giant ciliate host. ISME J 12:714–727.

14. Jäckle O, Seah BKB, Tietjen M, Leisch N, Liebeke M, Kleiner M, Berg JS, Gruber-Vodicka HR. 2019. Chemosynthetic symbiont with a drastically reduced genome serves as primary energy storage in the marine flatworm *Paracatenula*. Proc Natl Acad Sci 116:8505–8514.
15. Bailly X, Vinogradov S. 2005. The sulfide binding function of annelid hemoglobins: relic of an old biosystem? J Inorg Biochem 99:142–150.
16. Hildebrandt TM, Grieshaber MK. 2008. Three enzymatic activities catalyze the oxidation of sulfide to thiosulfate in mammalian and invertebrate mitochondria. FEBS J 275:3352–3361.
17. Grieshaber MK, Völkel S. 1998. Animal adaptations for tolerance and exploitation of poisonous sulfide. Annu Rev Physiol 33–53.
18. Powell MA, Somero GN. 1986. Adaptations to sulfide by hydrothermal vent animals: sites and mechanisms of detoxification and metabolism. Biol Bull 171:274–290.
19. Kraus DW, Doeller JE. 2004. Sulfide consumption by mussel gill mitochondria is not strictly tied to oxygen reduction: measurements using a novel polarographic sulfide sensor. J Exp Biol 207:3667–3679.
20. Ponnudurai R, Kleiner M, Sayavedra L, Petersen JM, Moche M, Otto A, Becher D, Takeuchi T, Satoh N, Dubilier N, Schweder T, Markert S. 2017. Metabolic and physiological interdependencies in the *Bathymodiolus azoricus* symbiosis. ISME J2016/11/02. 11:463–477.
21. Robidart JC, Roque A, Song P, Girguis PR. 2011. Linking hydrothermal geochemistry to organismal physiology: physiological versatility in *Riftia pachyptila* from sedimented and basalt-hosted vents. PLoS One 6:e21692.
22. Markert S, Gardebrecht A, Felbeck H, Sievert SM, Klose J, Becher D, Albrecht D, Thürmer A, Daniel R, Kleiner M, Hecker M, Schweder T. 2011. Status quo in physiological proteomics of the uncultured *Riftia pachyptila* endosymbiont. Proteomics 11:3106–3117.
23. Wilmot Jr. DB, Vetter RD. 1990. The bacterial symbiont from the hydrothermal vent tubeworm *Riftia pachyptila* is a sulfide specialist. Mar Biol 106:273–283.
24. Zal F, Lallier H, Green BN, Vinogradov SN. 1996. The multi-hemoglobin system of the hydrothermal vent tube worm *Riftia pachyptila*. II. Complete polypeptide chain composition investigated by maximum entropy analysis of mass spectra. J Biol Chem 271:8875–8881.
25. Zal F, Lallier FH, Wall JS, Vinogradov SN, Toulmond A. 1996. The multi-hemoglobin system of the hydrothermal vent tube worm *Riftia pachyptila*: I. Reexamination of the number and masses of its constituents. J Biol Chem 271:8869–8874.
26. Zal F, Leize E, Lallier FH, Toulmond A, Van Dorsselaer A, Childress J. 1998. S-sulfohemoglobin

and disulfide exchange: The mechanisms of sulfide binding by *Riftia pachyptila* hemoglobins. Proc Natl Acad Sci USA 95:8997–9002.

27. Flores JF, Fisher CR, Carney SL, Green BN, Freytag JK, Schaeffer SW, Royer Jr. WE. 2005. Sulfide binding is mediated by zinc ions discovered in the crystal structure of a hydrothermal vent tubeworm hemoglobin. Proc Natl Acad Sci U S A 102:2713–2718.
28. Hourdez S, Weber RE. 2005. Molecular and functional adaptations in deep-sea hemoglobins. J Inorg Biochem 99:130–141.
29. Sanchez S, Hourdez S, Lallier FH. 2007. Identification of proteins involved in the functioning of *Riftia pachyptila* symbiosis by Subtractive Suppression Hybridization. BMC Genomics 8:337.
30. Bailly X, Chabasse C, Hourdez S, Dewilde S, Martial S, Moens L, Zal F. 2007. Globin gene family evolution and functional diversification in annelids. FEBS J 274:2641–2652.
31. van der Land J, Nørrevang A. 1977. Structure and relationships of *Lamellibrachia* (Annelida, Vestimentifera). Det Kongelige Danske Videnskabernes Selskab, Biologiske Skrifter 21:3.
32. Hourdez S, Lallier FH, De Cian MC, Green BN, Weber RE, Toulmond A, Green BN, Weber RE, Toulmond A. 2000. Gas transfer system in *Alvinella pompejana* (Annelida Polychaeta, Terebellida): functional properties of intracellular and extracellular hemoglobins. Physiol Biochem Zool 73:365–373.
33. Hankeln T, Ebner B, Fuchs C, Gerlach F, Haberkamp M, Laufs TL, Roesner A, Schmidt M, Weich B, Wystub S, Saaler-Reinhardt S, Reuss S, Bolognesi M, De Sanctis D, Marden MC, Kiger L, Moens L, Dewilde S, Nevo E, Avivi A, Weber RE, Fago A, Burmester T. 2005. Neuroglobin and cytoglobin in search of their role in the vertebrate globin family. J Inorg Biochem 99:110–119.
34. Mangum CP. 1992. Physiological function of the hemerythrins, p. 173–192. *In* C.P., M (ed.), Blood and Tissue Oxygen Carriers. Advances in Comparative and Environmental Physiology, vol 13. Springer, Berlin, Heidelberg.
35. Vanin S, Negrisolo E, Bailly X, Bubacco L, Beltramini M, Salvato B. 2006. Molecular evolution and phylogeny of sipunculan hemerythrins. J Mol Evol 62:32–41.
36. Stenkamp RE. 1994. Dioxygen and hemerythrin. Chem Rev 94:715–726.
37. Sanchez S. 2007. Approche transcriptomique pour identifier des protéines impliquées dans le fonctionnement de la symbiose *Riftia pachyptila*. Dissertation. Univ. Pierre et Marie Curie.
38. Costa-Paiva EM, Whelan N V., Waits DS, Santos SR, Schrago CG, Halanych KM. 2017. Discovery and evolution of novel hemerythrin genes in annelid worms. BMC Evol Biol 17:85.

39. Coates CJ, Decker H. 2017. Immunological properties of oxygen-transport proteins: hemoglobin, hemocyanin and hemerythrin. Cell Mol Life Sci 74:293–317.

40. Wittenberg JB. 2007. On optima: The case of myoglobin-facilitated oxygen diffusion. Gene 398:156–161.

41. Bright M, Lallier FH. 2010. The biology of vestimentiferan tubeworms. Oceanogr Mar Biol 48:213–266.

42. Demuynck S, Bocquet-Muchembled B, Deloffre L, Grumiaux F, Leprêtre A. 2004. Stimulation by cadmium of myohemerythrin-like cells in the gut of the annelid *Nereis diversicolor*. J Exp Biol 207:1101–1111.

43. Wippler J, Kleiner M, Lott C, Gruhl A, Abraham PE, Giannone RJ, Young JC, Hettich RL, Dubilier N. 2016. Transcriptomic and proteomic insights into innate immunity and adaptations to a symbiotic lifestyle in the gutless marine worm *Olavius algarvensis*. BMC Genomics 17:942.

44. Seo J-K, Nam B-H, Go H-J, Jeong M, Lee K, Cho S-M, Lee I-A, Park NG. 2016. Hemerythrin-related antimicrobial peptide, msHemerycin, purified from the body of the lugworm, *Marphysa sanguinea*. Fish Shellfish Immunol 57:49–59.

45. Hahlbeck E, Pospesel MA, Zal F, Childress JJ, Felbeck H. 2005. Proposed nitrate binding by hemoglobin in *Riftia pachyptila* blood. Deep Res Part I Oceanogr Res Pap 52:1885–1895.

46. Sanchez S, Andersen AC, Hourdez S, Lallier FH. 2007. Identification, sequencing, and localization of a new carbonic anhydrase transcript from the hydrothermal vent tubeworm *Riftia pachyptila*. FEBS J 274:5311–5324.

47. De Cian M-C, Bailly X, Morales J, Strub J-M, Van Dorsselaer A, Lallier F. 2003. Characterization of carbonic anhydrases from *Riftia pachyptila*, a symbiotic invertebrate from deep-sea hydrothermal vents. PROTEINS Struct Funct Genet 51:327–339.

48. De Cian M-C, Andersen AC, Toullec J-Y, Biegala I, Caprais J-C, Shillito B, Lallier FH. 2003. Isolated bacteriocyte cell suspensions from the hydrothermal-vent tubeworm *Riftia pachyptila*, a potent tool for cellular physiology in a chemoautotrophic symbiosis. Mar Biol 142:141–151.

49. Felbeck H. 1985. CO_2 fixation in the hydrothermal vent tube worm *Riftia pachyptila* (Jones). Physiol Zool 58:272–281.

50. Felbeck H, Somero GN. 1982. Primary production in deep-sea hydrothermal vent organisms: roles of sulfide-oxidizing bacteria. TRENDS Biochem SCI 201–204.

51. Childress JJ, Arp AJ, Fisher Jr. CR. 1984. Metabolic and blood characteristics of the

hydrothermal vent tube-worm *Riftia pachyptila*. Mar Biol 83:109–124.

52. Malm S, Tiffert Y, Micklinghoff J, Schultze S, Joost I, Weber I, Horst S, Ackermann B, Schmidt M, Wohlleben W, Ehlers S, Geffers R, Reuther J, Bange FC. 2009. The roles of the nitrate reductase NarGHJI, the nitrite reductase NirBD and the response regulator GlnR in nitrate assimilation of *Mycobacterium tuberculosis*. Microbiology 155:1332–1339.
53. Liao L, Wankel SD, Wu M, Cavanaugh CM, Girguis PR. 2014. Characterizing the plasticity of nitrogen metabolism by the host and symbionts of the hydrothermal vent chemoautotrophic symbioses *Ridgeia piscesae*. Mol Ecol 23:1544–1557.
54. Sparacino-Watkins C, Stolz JF, Basu P. 2014. Nitrate and periplasmic nitrate reductases. Chem Soc Rev 43:676–706.
55. Hirayama C, Saito H, Konno K, Shinbo H. 1998. Purification and characterization of NADH-dependent glutamate synthase from the silkworm fat body (*Bombyx mori*). Insect Biochem Mol Biol 28:473–482.
56. Hirayama C, Konno K, Shinbo H. 1997. The pathway of ammonia assimilation in the silkworm, *Bombyx mori*. J Insect Physiol 43:959–964.
57. Scaraffia PY, Isoe J, Murillo A, Wells MA. 2005. Ammonia metabolism in *Aedes aegypti*. Insect Biochem Mol Biol 35:491–503.
58. Minic Z, Hervé G. 2003. Arginine metabolism in the deep sea tube worm *Riftia pachyptila* and its bacterial endosymbiont. J Biol Chem 278:40527–40533.
59. De Cian M-C, Regnault M, Lallier FH. 2000. Nitrogen metabolites and related enzymatic activities in the body fluids and tissues of the hydrothermal vent tubeworm *Riftia pachyptila*. J Exp Biol 203:2907–2920.
60. Fernandez IM, Silva M, Schuch R, Walker WA, Siber AM, Maurelli AT, McCormick BA. 2001. Cadaverine prevents the escape of *Shigella flexneri* from the phagolysosome: a connection between bacterial dissemination and neutrophil transepithelial signaling. J Infect Dis 184:743–753.
61. Zhang M, Borovikova L V, Wang H, Metz C, Tracey KJ. 1999. Spermine inhibition of monocyte activation and inflammation. Mol Med 5:595–605.
62. Igarashi K, Kashiwagi K. 2010. Modulation of cellular function by polyamines. Int J Biochem Cell Biol 42:39–51.
63. Wallace HM, Fraser A V., Hughes A. 2003. A perspective of polyamine metabolism. Biochem J 376:1–14.
64. Shah P, Swiatlo E. 2008. A multifaceted role for polyamines in bacterial pathogens. Mol

Microbiol 68:4–16.

65. Pflugfelder B, Cary SC, Bright M. 2009. Dynamics of cell proliferation and apoptosis reflect different life strategies in hydrothermal vent and cold seep vestimentiferan tubeworms. Cell Tissue Res 337:149–165.

66. Ferguson III JE, Wu Y, Smith K, Charles P, Powers K, Wang H, Patterson C. 2007. ASB4 is a hydroxylation substrate of FIH and promotes vascular differentiation via an oxygen-dependent mechanism. Mol Cell Biol 27:6407–6419.

67. Shen C, Powell-Coffman JA. 2003. Genetic analysis of hypoxia signaling and response in *C. elegans*. An New York Acad Sci 995:191–199.

68. Watanabe T, Fukuda I, China K, Isa Y. 2003. Molecular analyses of protein components of the organic matrix in the exoskeleton of two scleractinian coral species. Comp Biochem Physiol Part B 136:767–774.

69. Heath-Heckman EAC, Gillette AA, Augustin R, Gillette MX, Goldman WE, McFall-Ngai MJ. 2014. Shaping the microenvironment: evidence for the influence of a host galaxin on symbiont acquisition and maintenance in the squid-vibrio symbiosis. Environ Microbiol 16:3669–3682.

70. Ratzka C, Gross R, Feldhaar H. 2012. Endosymbiont tolerance and control within insect hosts. Insects 3:553–572.

71. Dabrowska K, Switała-Jelen K, Opolski A, Weber-Dabrowska B, Gorski A. 2005. Bacteriophage penetration in vertebrates. J Appl Microbiol 98:7–13.

72. Gardebrecht A, Markert S, Sievert SM, Felbeck H, Thürmer A, Albrecht D, Wollherr A, Kabisch J, Le Bris N, Lehmann R, Daniel R, Liesegang H, Hecker M, Schweder T. 2012. Physiological homogeneity among the endosymbionts of *Riftia pachyptila* and *Tevnia jerichonana* revealed by proteogenomics. ISME J 6:766–776.

73. Perez M, Juniper SK. 2016. Insights into symbiont population structure among three vestimentiferan tubeworm host species at eastern Pacific spreading centers. Appl Environ Microbiol 82:5197–5205.

74. Kádár E, Davis SA, Lobo-da-Cunha A. 2008. Cytoenzymatic investigation of intracellular digestion in the symbiont-bearing hydrothermal bivalve *Bathymodiolus azoricus*. Mar Biol 153:995–1004.

75. Caro A, Got P, Bouvy M, Troussellier M, Gros O. 2009. Effects of long-term starvation on a host bivalve (*Codakia orbicularis*, Lucinidae) and its symbiont population. Appl Environ Microbiol 75:3304–3313.

76. Johnson MA, Fernandez C. 2001. Bacterial symbiosis in *Loripes lucinalis* (Mollusca: Bivalvia) with comments on reproductive strategy. J Mar Biol Assoc United Kingdom 81:251–257.
77. Stewart FJ, Cavanaugh CM. 2006. Bacterial endosymbioses in *Solemya* (Mollusca: Bivalvia)—model systems for studies of symbiont-host adaptation. Antonie van Leeuwenhoek, Int J Gen Mol Microbiol 90:343–360.
78. Bright M, Bulgheresi S. 2010. A complex journey: transmission of microbial symbionts. Nat Rev Microbiol 8:218–230.
79. Nussbaumer AD, Fisher CR, Bright M. 2006. Horizontal endosymbiont transmission in hydrothermal vent tubeworms. Nature 441:345–348.
80. Kuwahara H, Yoshida T, Takaki Y, Shimamura S, Nishi S, Harada M, Matsuyama K, Takishita K, Kawato M, Uematsu K, Fujiwara Y, Sato T, Kato C, Kitagawa M, Kato I, Maruyama T. 2007. Reduced genome of the thioautotrophic intracellular symbiont in a deep-sea clam, *Calyptogena okutanii*. Curr Biol 17:881–886.
81. Rolando M, Sanulli S, Rusniok C, Gomez-Valero L, Bertholet C, Sahr T, Margueron R, Buchrieser C. 2013. *Legionella pneumophila* effector RomA uniquely modifies host chromatin to repress gene expression and promote intracellular bacterial replication. Cell Host Microbe 13:395–405.
82. Grininger M, Staudt H, Johansson P, Wachtveitl J, Oesterhelt D. 2009. Dodecin is the key player in flavin homeostasis of archaea. J Biol Chem 284:13068–13076.
83. Meissner B, Schleicher E, Weber S, Essen L-O. 2007. The dodecin from *Thermus thermophilus*, a bifunctional cofactor storage protein. J Biol Chem 282:33142–33154.
84. Massey V. 1994. Activation of molecular oxygen by flavins and flavoproteins. J Biol Chem 269:22459–22462.
85. Bourdeaux F, Hammer CA, Vogt S, Schweighöfer F, Nöll G, Wachtveitl J, Grininger M. 2018. Flavin storage and sequestration by *Mycobacterium tuberculosis* dodecin. ACS Infect Dis 4:1082–1092.
86. Wozniak RAF, Waldor MK. 2009. A toxin-antitoxin system promotes the maintenance of an integrative conjugative element. PLoS Genet 5:e1000439.
87. Lazzaroni J-C, Dubuisson J-F, Vianney A. 2002. The Tol proteins of *Escherichia coli* and their involvement in the translocation of group A colicins. Biochimie 84:391–397.
88. Yang S-C, Lin C-H, Sung CT, Fang J-Y. 2014. Antibacterial activities of bacteriocins: application in foods and pharmaceuticals. Front Microbiol 5:241.
89. Scott KM, Boller AJ, Dobrinski KP, Le Bris N. 2012. Response of hydrothermal vent

vestimentiferan *Riftia pachyptila* to differences in habitat chemistry. Mar Biol 159:435–442.

90. Childress JJ, Fisher CR, Favuzzi JA, Kochevar RE, Sanders NK, Alayse AM. 1991. Sulfide-driven autotrophic balance in the bacterial symbiont-containing hydrothermal vent tubeworm, *Riftia pachyptila* Jones. Biol Bull 180:135–153.

91. Girguis PR, Lee RW, Desaulniers N, Childress JJ, Pospesel M, Felbeck H, Zal F. 2000. Fate of nitrate acquired by the tubeworm *Riftia pachyptila*. Appl Environ Microbiol 66:2783–2790.

92. Schramm M, Wiegmann K, Schramm S, Gluschko A, Herb M, Utermöhlen O, Krönke M. 2014. Riboflavin (vitamin B_2) deficiency impairs NADPH oxidase 2 (Nox2) priming and defense against *Listeria monocytogenes*. Eur J Immunol 44:728–741.

93. Zeng WB, Chen WB, Yan QP, Lin GF, Qin YX. 2016. Hemerythrin is required for *Aeromonas hydraphlia* to survive in the macrophages of *Anguilla japonica*. Genet Mol Res 15:gmr.15028074.
