# Supplementary material for: Host-Microbe Interactions in the Chemosynthetic Riftia pachyptila Symbiosis
Source: mBio. 2019 Dec 17;10(6):e02243-19. doi: 10.1128/mBio.02243-19 (PMC6918071; doi:10.1128/mBio.02243-19)
Supplement: TABLE S2 [file mBio.02243-19-st002.pdf]

Supp. Table S2: Domains and protein families with a putative role in host-symbiont interactions. The domains and protein families listed here were included in the comparisons in Figure 5 and Supp. Figure S5, which show the percentage of the respective protein groups in the *Riftia* symbiont metagenome and in metagenomes of other symbiotic and free-living organisms. % bacterial, total number bacterial: Percentage and total number of bacterial species in which this domain is found in the SMART database (January 2019).

**Domain name**	**Pfam/SMART annotation**	**% bacterial (total number bacterial)**	**Literature/comment**
Alpha-2-macroglobulin	alpha-2-macroglobulin family (A2M), including N-terminal MG1 domain	A2M: 42.05% (2057)	A2Ms: protease inhibitors which are important for eukaryotic innate immunity, if present in prokaryotes apparently fulfill a similar role, e.g. protection against host proteases (1)
ANAPC	Anaphase-promoting complex subunits	APC2: 0	Ubiquitin ligase, important for cell cycle control in eukaryotes (2)<br>Bacterial proteins might interact with ubiquitination pathways in the host (3)
Ankyrin	Ankyrin repeats	10.88% (8348)	Mediate protein-protein interactions without sequence specificity (4)<br>Sponge symbiont ankyrin-repeat proteins inhibit amoebal phagocytosis (5)<br>Present in sponge microbiome metatranscriptomes, putative role in symbiont-host interactions (6)<br>Present in obligate intracellular amoeba symbiont *Candidatus* Amoebophilus asiaticus genome, probable function in interactions with the host (7)
Armadillo	Armadillo repeats	0.83% (67)	Eukaryotic armadillo repeats are involved in protein-protein interactions, e.g. in intracellular signaling and cytoskeletal organization (8)

Domain name	Pfam/SMART annotation	% bacterial (total number bacterial)	Literature/comment
Cadherin	Cadherin domains, Cadherin-homologous	CA: 6.4% (956)<br>CADG: 47.77% (739)	Calcium-dependent cell-cell adhesion, tissue morphogenesis in animals (9)<br>Present in sponge microbiome metatranscriptomes, putative role in symbiont-host interactions (6)<br>Putative mediation of protein interactions and cell-cell adhesion in the marine bacterium *Saccharophagus degradans*; most cadherin-containing prokaryotes are aquatic (10)
Coatomer	coatomer complex units	NA	Formation of vesicles for intracellular transport (11)
coiled-coil	coiled-coil domains	NA	Structural domain in all three domains of life, in proteins of diverse functions (12)<br>Virulence effectors secreted by type III secretion systems of pathogenic bacteria often contain coiled-coil domains, which e.g. could interact with host signaling pathways (13)
Dynamin	Dynamin family	DYNc: 0	Large GTPases which in eukaryotes are important for vesicle scission and lipid tabulation and fission (14)<br>Conserved in many bacterial genomes, cellular role of bacterial dynamins not clear, could be involved in cytokinesis under osmotic stress (15)
F-box	ubiquitin interacting	0.42% (92)	Present in obligate intracellular amoeba symbiont *Candidatus* Amoebophilus asiaticus genome, could interact with host ubiquitination pathways (7)<br>Bacterial proteins might interact with ubiquitination pathways in the host (3)
FG-GAP	Extracellular repeat in alpha integrins	NA	*Leptospira* FG-GAP proteins could be involved in interaction with host tissues (16)<br>Also see Integrin

**Domain name**	**Pfam/SMART annotation**	**% bacterial (total number bacterial)**	**Literature/comment**
Fibronectin	Fibronectin (FN) and FN-like	FN1: 0<br>FN2: 0<br>FN3: 24.19% (11706)	Present in sponge microbiome metatranscriptomes, putative role in symbiont-host interactions (6)
GIDE	E3 ubiquitin ligase	NA	Bacterial proteins might interact with ubiquitin pathways in the host (3)
HAT	Half A TPR repeat	2.00% (86)	RNA and peptide binding motif, involved in RNA metabolism, related to TPR repeat, highly conserved in eukaryotes, almost all HAT proteins are found in nucleus (17)
He_PIG	putative immunoglobulin	NA	See Immunoglobulin
HEAT	HEAT and HEAT-like repeats	NA	Found in eukaryotic proteins with different functions, involved in protein-protein interactions, related to armadillo and ankyrin repeats (18)
Host_attach	bacterial attachment to host cells	NA	Required for attachment to host cells (19)
HYR	hyalin repeat domain	NA	Belongs to immunoglobulin-like fold, probably involved in cell adhesion in eukaryotes (20)
Immuno-globulin	Immunoglobulin, immunoglobulin-like	1.24% (624)	Present in functionally diverse proteins, involved in molecular recognition, in pathogenic *E. coli* strains important for host cell infection, components of adhesins and other proteins (21)<br>Bacterial surface proteins could be involved in recognition between symbionts and host

Domain name	Pfam/SMART annotation	% bacterial (total number bacterial)	Literature/comment
Integrin	Integrin alpha and beta subunits	Int_alpha: 26.37% (1071)<br>INB: 1.2% (16)	Integrins are important for cell adhesion and signaling in metazoans, also present in apusozoans; uncommon in prokaryotes (22)<br>Bacterial structures not found to be full-length integrins, but full-length beta-propeller domains are present, function unknown (23)<br>Could be involved in interaction with host tissues in *Leptospira* (16)
Laminin	Laminin domains	LamNT: 0.84% (22)<br>LamB: 0<br>LamG: 6.49% (544)<br>EGF_Lam: 0	Important constituent of basement membranes in animals (24)
Lectin	lectins	Jacalin: 7.49% (96)<br>Gal-bind_lectin: 0.08% (2)<br>B_lectin: 5.58% (219)<br>CLECT: 1.54% (263)<br>GLECT: 0.13% (3)	Carbohydrate-binding proteins, involved in immune system reaction by pathogen recognition and aggregation in vertebrates and invertebrates (25)<br>Virulence factors in pathogens (26)
LPG_synthase_TM	Lysylphosphatidylglycerol synthase TM region	NA	Lysylphosphatidylglycerol synthase conveys resistance of *Staphylococcus aureus* against cationic antimicrobial peptides by modifying the cell membrane (27)
LppC	bacterial outer membrane antigens	NA	Bacterial surface proteins could be involved in recognition between symbionts and host
LRR	leucine-rich repeats	LRR: 6.08% (3688)<br>LRR_TYP: 5.05% (1263)	Present in obligate intracellular amoeba symbiont *Candidatus* Amoebophilus asiaticus genome, putative function in interactions with the host (7)
LTD	lamin tail domain	NA	Nuclear lamins are part of the nuclear lamina, probably also involved in DNA interaction (28)

Domain name	Pfam/SMART annotation	% bacterial (total number bacterial)	Literature/comment
LTXXQ	LTXXQ motif family protein	NA	Motif in CpxP, a member of the two-component signal transduction Cpx pathway, which reacts to cell envelope stresses and misfolded proteins (29)
MIF	macrophage migration inhibitory factor	NA	Involved in innate immunity of vertebrates and probably of invertebrates, as well as adaptive immunity of vertebrates, also found in plants and a cyanobacterium with unclear function (30)
NARP1	NMDA receptor-regulated protein 1	NA	Eukaryotic domain with similarity to cell cycle regulating yeast acetyltransferase (Pfam)
NHL	NCL-1, HT2A and Lin-41, similarity to WD repeat	NA	Present in sponge microbiome metatranscriptomes, putative role in symbiont-host interactions (6)
Nipsnap	NIPSNAP	NA	Could be involved in vesicular trafficking (31)<br>Present in sponge microbiome metatranscriptomes, putative role in symbiont-host interactions (6)
PQQ, PQQ-like	PQQ enzyme repeat	63.42% (6647)	Beta-propeller repeat in enzymes using pyrollo-quinoline quinone (PQQ) as prosthetic group (Pfam)<br>Present in sponge microbiome metatranscriptomes, putative role in symbiont-host interactions (6)
Sel1	TPR subfamily	61.04% (10391)	Present in sponge microbiome metatranscriptomes, putative role in symbiont-host interactions (6)<br>Present in obligate intracellular amoeba symbiont *Candidatus* Amoebophilus asiaticus genome, probable function in interactions with the host (7)
SNARE, IncA	SNARE domain, IncA protein	t_SNARE: 0.07% (4)	Intracellular bacteria can use SNARE-like proteins (including IncA) to block host SNARE-mediated membrane fusion and thereby endocytosis (32)

**Domain name**	**Pfam/SMART annotation**	**% bacterial (total number bacterial)**	**Literature/comment**
TIR	Toll-interleukin-receptor	1.04% (47)	Protein-protein interaction domain in innate immune system proteins of plants and animals, present in pathogenic and non-pathogenic bacteria, TIR domain-containing proteins of pathogens can interfere with host immune system (33)
TPR	tetratricopeptide repeats	51.13% (33476)	Present in sponge microbiome metatranscriptomes, putative role in symbiont-host interactions (6)<br>Present in obligate intracellular amoeba symbiont *Candidatus* Amoebophilus asiaticus genome, probable function in interactions with the host (7)<br>TPR-containing bacterial proteins can have impact on phagocytosis of bacteria by amoebae (34)
TSP	thrombospondins	TSP1: 0.15% (16)<br>TSPN: 0.18% (6)	Extracellular glycoproteins in animals, involved in cell attachment in vertebrates (35)
TTL	tubulin-tyrosine ligase family	NA	Tubulin posttranslational modification (tyrosination), essential for cell development (36)
U-box	ubiquitin interacting	0.44% (19)	Present in obligate intracellular amoeba symbiont *Candidatus* Amoebophilus asiaticus genome, could interact with host ubiquitination pathways (7)<br>Bacterial proteins might interact with ubiquitination pathways in the host (3)
UB activating E1	Domain of ubiquitin activating E1 family enzymes	UBA_E1_c: 0	Bacterial proteins might interact with ubiquitination pathways in the host (3)
UBA	ubiquitin associated domain	0.61% (48)	Bacterial proteins might interact with ubiquitination pathways in the host (3)

Domain name	Pfam/SMART annotation	% bacterial (total number bacterial)	Literature/comment
VIT	Vault protein Inter-alpha-Trypsin domain	0	Conserved domain in the inter-alpha-trypsin inhibitor heavy chain family, one of the precursor proteins of inter-alpha-trypsin inhibitors, which are protease inhibitors and involved in stabilization of the extracellular matrix (37)
VWA	Von Willebrand factor type A domain	47.46% (20119)	Domain involved in eukaryotes in protein-protein interactions, multiprotein complexes, extracellular matrix, cell adhesion, various intracellular functions; prokaryotic proteins mostly not well characterized, characterized bacterial proteins with different functions; some prokaryotic VWA proteins possibly acquired via horizontal gene transfer (38)
WD40, PD40	WD40, WD40-like repeats	WD40: 5.54% (5471)	WD40 repeat proteins are highly abundant proteins in eukaryotes, involved in various functions; comparatively rare in prokaryotes, here putatively also involved in different functions, enriched in Cyanobacteria and Planctomycetes (39)

References

1. Wong SG, Dessen A. 2014. Structure of a bacterial α2-macroglobulin reveals mimicry of eukaryotic innate immunity. Nat Commun 5:4917.
2. Peters J-M. 2006. The anaphase promoting complex/cyclosome: a machine designed to destroy. Nat Rev Mol Cell Biol 7:644–656.
3. Rytkönen A, Holden DW. 2007. Bacterial interference of ubiquitination and deubiquitination. Cell Host Microbe 1:13–22.
4. Li J, Mahajan A, Tsai M-D. 2006. Ankyrin repeat: a unique motif mediating protein-protein interactions. Biochemistry 45:15168–15178.
5. Nguyen MTHD, Liu M, Thomas T. 2014. Ankyrin-repeat proteins from sponge symbionts modulate amoebal phagocytosis. Mol Ecol 23:1635–1645.
6. Díez-Vives C, Moitinho-Silva L, Nielsen S, Reynolds D, Thomas T. 2017. Expression of eukaryotic-like protein in the microbiome of sponges. Mol Ecol 26:1432–1451.
7. Schmitz-Esser S, Tischler P, Arnold R, Montanaro J, Wagner M, Rattei T, Horn M. 2010. The genome of the amoeba symbiont "*Candidatus* Amoebophilus asiaticus" reveals common mechanisms for host cell interaction among amoeba-associated bacteria. J Bacteriol 192:1045–1057.
8. Coates JC. 2003. Armadillo repeat proteins: beyond the animal kingdom. Trends Cell Biol 13:463–471.
9. Koch AW, Manzur KL, Shan W. 2004. Structure-based models of cadherin-mediated cell adhesion: the evolution continues. Cell Mol Life Sci 61:1884–1895.
10. Fraiberg M, Borovok I, Weiner RM, Lamed R. 2010. Discovery and characterization of cadherin domains in *Saccharophagus degradans* 2-40. J Bacteriol 192:1066–1074.
11. Wang S, Zhai Y, Pang X, Niu T, Ding Y-H, Dong M-Q, Hsu VW, Sun Z, Sun F. 2016. Structural characterization of coatomer in its cytosolic state. Protein Cell 7:586–600.
12. Truebestein L, Leonard TA. 2016. Coiled-coils: the long and short of it. BioEssays 38:903–916.
13. Delahay RM, Frankel G. 2002. Coiled-coil proteins associated with type III secretion systems: a versatile domain revisited. Mol Microbiol 45:905–916.
14. Praefcke GJK, McMahon HT. 2004. The dynamin superfamily: universal membrane tubulation and fission molecules? Nat Rev Mol Cell Biol 5:133–147.
15. Bramkamp M. 2012. Structure and function of bacterial dynamin-like proteins. Biol Chem 393:1203–1214.
16. Chou LF, Chen YT, Lu CW, Ko YC, Tang CY, Pan MJ, Tian YC, Chiu CH, Hung CC, Yang CW. 2012. Sequence of *Leptospira santarosai* serovar Shermani genome and prediction of virulence-associated genes. Gene 511:364–370.
17. Hammani K, Cook WB, Barkan A. 2012. RNA binding and RNA remodeling activities of the half-a-tetratricopeptide (HAT) protein HCF107 underlie its effects on gene expression. Proc Natl Acad Sci 109:5651–5656.
18. Yoshimura SH, Hirano T. 2016. HEAT repeats – versatile arrays of amphiphilic helices working in crowded environments? J Cell Sci 129:3963–3970.
19. Matthysse AG, Yarnall H, Boles SB, McMahan S. 2000. A region of the *Agrobacterium tumefaciens* chromosome containing genes required for virulence and attachment to host cells. Biochim Biophys Acta 1490:208–212.

20. Callebaut I, Gilgès D, Vigon I, Mornon J. 2000. HYR, an extracellular module involved in cellular adhesion and related to the immunoglobulin-like fold. Protein Sci 9:1382–1390.
21. Bodelón G, Palomino C, Fernández LÁ. 2013. Immunoglobulin domains in Escherichia coli and other enterobacteria: From pathogenesis to applications in antibody technologies. FEMS Microbiol Rev 37:204–250.
22. Sebé-Pedrós A, Roger AJ, Lang FB, King N, Ruiz-Trillo I. 2010. Ancient origin of the integrin-mediated adhesion and signaling machinery. Proc Natl Acad Sci USA 107:10142–10147.
23. Chouhan B, Denesyuk A, Heino J, Johnson MS, Denessiouk K. 2011. Conservation of the human integrin-type beta-propeller domain in bacteria. PLoS One 6:e25069.
24. Sasaki T, Fässler R, Hohenester E. 2004. Laminin: the crux of basement membrane assembly. J Cell Biol 164:959–963.
25. Fujita T. 2002. Evolution of the lectin-complement pathway and its role in innate immunity. Nat Rev Immunol 2:346–353.
26. Imberty A, Wimmerová M, Mitchell EP, Gilboa-Garber N. 2004. Structures of the lectins from *Pseudomonas aeruginosa*: insights into the molecular basis for host glycan recognition. Microbes Infect 6:221–228.
27. Staubitz P, Neumann H, Schneider T, Wiedemann I, Peschel A. 2004. MprF-mediated biosynthesis of lysylphosphatidylglycerol, an important determinant in staphylococcal defensin resistance. FEMS Microbiol Lett 231:67–71.
28. Stuurman N, Heins S, Aebi U. 1998. Nuclear lamins: their structure, assembly, and interactions. J Struct Biol 122:42–66.
29. Zhou X, Keller R, Volkmer R, Krauss N, Scheerer P, Hunke S. 2011. Structural basis for two-component system inhibition and pilus sensing by the auxiliary CpxP protein. J Biol Chem 286:9805–9814.
30. Sparkes A, De Baetselier P, Roelants K, De Trez C, Magez S, Van Ginderachter JA, Raes G, Bucala R, Stijlemans B. 2017. The non-mammalian MIF superfamily. Immunobiology 222:858–867.
31. Lee AH, Zareei MP, Daefler S. 2002. Identification of a NIPSNAP homologue as host cell target for *Salmonella* virulence protein SpiC. Cell Microbiol 4:739–750.
32. Paumet F, Wesolowski J, Garcia-Diaz A, Delevoye C, Aulner N, Shuman HA, Subtil A, Rothman JE. 2009. Intracellular bacteria encode inhibitory SNARE-like proteins. PLoS One 4:e7375.
33. Ve T, Williams SJ, Kobe B. 2015. Structure and function of Toll/interleukin-1 receptor/resistance protein (TIR) domains. Apoptosis 20:250–261.
34. Reynolds D, Thomas T. 2016. Evolution and function of eukaryotic-like proteins from sponge symbionts. Mol Ecol 25:5242–5253.
35. Adams JC, Lawler J. 2004. The thrombospondins. Int J Biochem Cell Biol 36:961–968.
36. Prota AE, Magiera MM, Kuijpers M, Bargsten K, Frey D, Wieser M, Jaussi R, Hoogenraad CC, Kammerer RA, Janke C, Steinmetz MO. 2013. Structural basis of tubulin tyrosination by tubulin tyrosine ligase. J Cell Biol 200:259–270.
37. Himmelfarb M, Klopocki E, Grube S, Staub E, Klaman I, Hinzmann B, Kristiansen G, Rosenthal A, Dürst M, Dahl E. 2004. *ITIH5*, a novel member of the inter-α-trypsin inhibitor heavy chain family is downregulated in breast cancer. Cancer Lett 204:69–77.
38. Whittaker CA, Hynes RO. 2002. Distribution and evolution of von Willebrand/integrin A domains: widely dispersed domains with roles in cell adhesion and elsewhere. Mol Biol Cell 13:3369–3387.
39. Hu XJ, Li T, Wang Y, Xiong Y, Wu X-H, Zhang D-L, Ye Z-Q, Wu Y-D. 2017. Prokaryotic and highly-repetitive WD40 proteins: a systematic study. Nat Sci Reports 7:10585.
