# Supplementary material for: Host-Microbe Interactions in the Chemosynthetic Riftia pachyptila Symbiosis
Source: mBio. 2019 Dec 17;10(6):e02243-19. doi: 10.1128/mBio.02243-19 (PMC6918071; doi:10.1128/mBio.02243-19)
Supplement: TABLE S3 [file mBio.02243-19-st003.pdf]

Supp. Table S3: Transcriptome completeness for the four different *Riftia* transcriptome assemblies based on the BUSCO eukaryote and metazoan datasets. C: complete BUSCOs, S: complete and single-copy BUSCOs, D: complete and duplicated BUSCOs, F: fragmented BUSCOs, M: missing BUSCOs. Assembly 4 was used for all further analyses.

	**Completeness [%]**	
**Assembly**	*Eukaryota*	*Metazoa*
**1**	C: 99.0 [S: 32.3, D: 66.7], F: 1.0, M: 0.0	C: 98.3 [S: 31.4, D: 66.9], F: 0.9, M: 0.8
**2**	C: 99.0 [S: 33.0, D: 66.0], F: 0.7, M: 0.3	C: 98.3 [S: 32.5, D: 65.8], F: 0.9, M: 0.8
**3**	C: 99.0 [S: 36.0, D: 63.0], F: 1.0, M: 0.0	C: 98.4 [S: 39.7, D: 58.7], F: 0.9, M: 0.7
**4**	C: 99.3 [S: 33.0, D: 66.3], F: 0.7, M: 0.0	C: 98.8 [S: 37.1, D: 61.7], F: 0.7, M: 0.5
