# Supplementary material for: Host-Microbe Interactions in the Chemosynthetic Riftia pachyptila Symbiosis
Source: mBio. 2019 Dec 17;10(6):e02243-19. doi: 10.1128/mBio.02243-19 (PMC6918071; doi:10.1128/mBio.02243-19)
Supplement: TABLE S4 [file mBio.02243-19-st004.pdf]

Supp. Table S4: Tools used to characterize *Riftia* host and symbiont proteins included in the combined *Riftia* host and symbiont database used in this study.

**Tool**	**Used for host/ symbiont (Sym) proteins**	**Prediction**	**Reference**
Pfam*	Host, Sym	functional annotation	(1)
TIGRFAM*	Host, Sym	functional annotation	(2)
KEGG*	Host, Sym	functional annotation	(3)
eggNOG*	Host, Sym	functional annotation	(4)
BlastKoala	Host, Sym	functional annotation	(5)
Phobius	Host, Sym	signal peptides and transmembrane domains	(6)
SignalP	Host, Sym	signal peptides	(7)
TMHMM	Host, Sym	transmembrane helices	(8)
CELLO	Host, Sym	subcellular localization	(9)
PSortb	Sym	subcellular localization	(10)
SecretomeP	Sym	non-classical secretion signals	(11)
Lipo	Sym	lipoproteins	(12)
BOMP	Sym	β-barrel outer membrane proteins	(13)
TargetP	Host	subcellular localization	(14)
WoLF PSORT	Host	subcellular localization	(15)
DeepLoc	Host	subcellular localization	(16)
SLP-Local	Host	subcellular localization	(17)

* Metaerg (https://sourceforge.net/projects/metaerg/) was used to query these databases

References

1. El-Gebali S, Mistry J, Bateman A, Eddy SR, Luciani A, Potter SC, Qureshi M, Richardson LJ, Salazar GA, Smart A, Sonnhammer ELL, Hirsh L, Paladin L, Piovesan D, Tosatto SCE, Finn RD. 2019. The Pfam protein families database in 2019. Nucleic Acids Res 47:D427–D432.
2. Haft DH, Loftus BJ, Richardson DL, Yang F, Eisen JA, Paulsen IT, White O. 2001. TIGRFAMs: a protein family resource for the functional identification of proteins. Nucleic Acids Res 29:41–43.
3. Kanehisa M, Furumichi M, Tanabe M, Sato Y, Morishima K. 2017. KEGG: new perspectives on genomes, pathways, diseases and drugs. Nucleic Acids Res 45:D353–D361.
4. Huerta-Cepas J, Szklarczyk D, Forslund K, Cook H, Heller D, Walter MC, Rattei T, Mende DR, Sunagawa S, Kuhn M, Jensen LJ, von Mering C, Bork P. 2016. eggNOG 4.5: a hierarchical orthology framework with improved functional annotations for eukaryotic, prokaryotic and viral sequences. Nucleic Acids Res 44:D286–D293.
5. Kanehisa M, Sato Y, Morishima K. 2016. BlastKOALA and GhostKOALA: KEGG tools for functional characterization of genome and metagenome sequences. J Mol Biol 428:726–731.
6. Käll L, Krogh A, Sonnhammer ELL. 2007. Advantages of combined transmembrane topology and signal peptide prediction — the Phobius web server. Nucleic Acids Res 35:W429–W432.
7. Nielsen H. 2017. Predicting secretory proteins with SignalP, p. 59–73. *In* Kihara, D (ed.), Protein Function Prediction. Methods in Molecular Biology, vol 1611. Humana Press, New York, NY.
8. Krogh A, Larsson B, von Heijne G, Sonnhammer ELL. 2001. Predicting transmembrane protein topology with a hidden Markov model: application to complete genomes. J Mol Biol 305:567–580.
9. Yu C-S, Chen Y-C, Lu C-H, Hwang J-K. 2006. Prediction of protein subcellular localization. PROTEINS Struct Funct Bioinforma 64:643–651.
10. Yu NY, Wagner JR, Laird MR, Melli G, Rey S, Lo R, Dao P, Sahinalp SC, Ester M, Foster LJ, Brinkman FSL. 2010. PSORTb 3.0: improved protein subcellular localization prediction with refined localization subcategories and predictive capabilities for all prokaryotes. Bioinformatics 26:1608–1615.
11. Bendtsen JD, Kiemer L, Fausbøll A, Brunak S. 2005. Non-classical protein secretion in bacteria. BMC Microbiol 13:58.
12. Berven FS, Karlsen OA, Straume AH, Flikka K, Murrell JC, Fjellbirkeland A, Lillehaug JR, Eidhammer I, Jensen HB. 2006. Analysing the outer membrane subproteome of *Methylococcus capsulatus* (Bath) using proteomics and novel biocomputing tools. Arch Mircrobiol 184:362–377.
13. Berven FS, Flikka K, Jensen HB, Eidhammer I. 2004. BOMP: a program to predict integral β-barrel outer membrane proteins encoded within genomes of Gram-negative bacteria. Nucleic Acids Res 32:W394–W399.
14. Emanuelsson O, Nielsen H, Brunak S, von Heijne G. 2000. Predicting subcellular localization of proteins based on their N-terminal amino acid sequence. J Mol Biol 300:1005–1016.
15. Horton P, Park K-J, Obayashi T, Fujita N, Harada H, Adams-Collier C, Nakai K. 2007. WoLF PSORT: protein localization predictor. Nucleic Acids Res 35:W585–W587.
16. Almagro Armenteros JJ, Sønderby CK, Sønderby SK, Nielsen H, Winther O. 2017. DeepLoc: prediction of protein subcellular localization using deep learning. Bioinformatics 33:3387–3395.
17. Matsuda S, Vert J-P, Saigo H, Ueda N, Toh H, Akutsu T. 2005. A novel representation of protein sequences for prediction of subcellular location using support vector machines. Protein Sci 14:2804–2813.
